# Identification and Antifungal Susceptibility Analysis of *Stephanoascus ciferrii* Complex Species Isolated From Patients With Chronic Suppurative Otitis Media

**DOI:** 10.3389/fmicb.2021.680060

**Published:** 2021-07-21

**Authors:** Penghao Guo, Zhongwen Wu, Pingjuan Liu, Yili Chen, Kang Liao, Yaqin Peng, Yuting He

**Affiliations:** Department of Clinical Laboratory, The First Affiliated Hospital, Sun Yat-sen University, Guangzhou, China

**Keywords:** *Stephanoascus ciferrii* complex, chronic suppurative otitis media, antifungal susceptibility analysis, MALDI-TOF MS, internal transcribed spacer region sequencing

## Abstract

**Background:**

*Stephanoascus ciferrii* is a heterothallic ascomycetous yeast-like fungus. Recently, the concept of *S. ciferrii* complex has been proposed and it consists of *S. ciferrii*, *Candida allociferrii*, and *Candida mucifera*. We aimed to identify 32 strains of *S. ciferrii* complex isolated from patients with chronic suppurative otitis media (CSOM) at the species level and analyze the morphology and antifungal susceptibility profiles of the three species.

**Method:**

The sequencing of the internal transcribed spacer (ITS) region and matrix-assisted laser desorption ionization time-of-flight mass spectrometry (MALDI-TOF MS) were used to identify *S. ciferrii* complex species. The SARAMIS software was used for cluster analysis of the mass spectra. All the strains were cultured on Sabouraud dextrose agar (SDA) and CHROM plates for 7 days. In the meantime, colonies of the 32 strains went through Gram staining. The Sensititre YeastOne YO10 colorimetric panel was used for the antifungal susceptibility analysis.

**Results:**

There were 10 strains of *C. allociferrii* (31.25%), six strains of *C. mucifera* (18.75%), and 16 strains of *S. ciferrii* (50%) in the 32 strains of *S. ciferrii* complex according to the sequencing of the ITS region. MALDI-TOF MS could identify *S. ciferrii* but showed no results for *C. allociferrii* and *C. mucifera*. The cluster analysis of the mass spectra by SARAMIS indicated that the MALDI-TOF MS could distinguish the three species. The morphology characteristics of the three species were similar. As for antifungal susceptibility, *S. ciferrii* and *C. mucifera* tended to have high fluconazole MICs compared with *C. allociferrii*. *C. mucifera* and *C. allociferrii* had relatively low flucytosine MICs while *S. ciferrii* owned high flucytosine MICs. Besides, *C. mucifera* tended to have a higher MIC value than *S. ciferrii* for amphotericin B and *C. allociferrii* for anidulafungin, micafungin, and caspofungin.

**Conclusion:**

The antifungal susceptibility profiles of the three species of *S. ciferrii* complex had their own characteristics. Besides, more mass spectra of *C. allociferrii* and *C. mucifera* are needed to construct the reference database for *S. ciferrii* complex species, enabling MALDI-TOF MS to identify *S. ciferrii* complex at species level.

## Introduction

*Stephanoascus ciferrii* (also called *Candida ciferrii* or *Trichomonascus ciferrii*) is a heterothallic ascomycetous yeast-like fungus, which is a teleomorph of *Candida ciferrii* (de Gentile et al., 1991; Warren et al., 2018). It has been reported to cause various human infections such as endophthalmitis, intraorbital abscess, systemic mycosis, and otitis media (Soki et al., 2010, 2015; Agin et al., 2011; Danielescu et al., 2017). Due to the development of molecular biology techniques for the identification of fungi, more and more species have been discovered. Recently, the concept of microbial complexes is applied. Microbial complex refers to a group of pathogens which are phenotypically indistinguishable but different at the genetic level, such as *Acinetobacter calcoaceticus-baumannii* complex and *Candida parapsilosis* complex (Mancilla-Rojano et al., 2020; da Silva et al., 2021). By sequencing of the 18S rRNA gene, Kumiko Ueda-Nishimura and Kozaburo Mikata divided *S. ciferrii* into three groups and proposed the *S. ciferrii* complex, which consists of *S. ciferrii*, *Candida allociferrii*, and *Candida mucifera* (Ueda-Nishimura and Mikata, 2002). Usually, the widely used VITEK 2 yeast identification system (bioMérieux, Marcy-l’Étoile, France) just identified the microorganism as *S. ciferrii* complex and it does not identify the complex at species level (Dave et al., 2018). Also, many clinicians do not distinguish the three species of *S. ciferrii* complex and they just take it as *S. ciferrii*. However, as for a rare kind of opportunistic pathogenic fungus, identifying *S. ciferrii* complex at species level is of great importance in epidemiological studies and the management of empirical antifungal therapy. Recently, matrix-assisted laser desorption ionization time-of-flight mass spectrometry (MALDI-TOF MS) has revolutionized microbial diagnostics (Cuenod et al., 2021). Because of its minimal hands-on and turnaround time, low costs, and high accuracy, MALDI-TOF MS has been widely used in the identification of bacterial and fungi species clinically (Angeletti and Ciccozzi, 2019; Cuenod et al., 2021).

Chronic suppurative otitis media (CSOM) is a chronic inflammation, characterized by repeated otorrhea *via* tympanic membrane perforation and involving polymicrobial infection of the middle ear and mastoid cavity (Bhutta et al., 2020). Usually, the common pathogens causing CSOM are *Staphylococcus aureus*, *Pseudomonas aeruginosa*, and coagulase-negative *Staphylococcus* (Xu et al., 2020). However, the isolation rate of *S. ciferrii* complex has increased recently in our hospital. In our study, we collected 32 strains of *S. ciferrii* complex, which were isolated from patients with CSOM and identified by the VITEK 2 yeast identification system. The sequencing of the internal transcribed spacer (ITS) region was used to identify them at species level. Then, the morphology, antifungal susceptibility, and mass spectra from MALDI-TOF MS of the 32 strains of *S. ciferrii* complex were analyzed and compared, providing experimental evidence and clinical experience for the diagnosis and treatment of the rare fungi at species level.

## Materials and Methods

### Materials

Thirty-two yeast strains were isolated from the middle ear secretions of patients with CSOM at The First Affiliated Hospital of Sun Yat-sen University from May 2019 to February 2020 and identified as *S. ciferrii* complex by the VITEK 2 yeast identification system (bioMérieux, France). Our study was approved by the institutional review board of The First Affiliated Hospital.

### The Sequencing and Alignment of the Internal Transcribed Spacer Region

The strains were cultured at 28°C with shaking in test tubes containing 5 ml YPD broth overnight. One milliliter of overnight yeast culture was centrifuged, and yeast precipitations were collected for the DNA extraction. The Yeast Genomic DNA Rapid Extraction Kit (Sangon Biotech, Shanghai, China) was used for the DNA extraction of collected yeast strains. All experimental operations and experimental conditions were performed according to the manufacturer’s instructions. The sequences of the ITS region rDNA were amplified using the universal primers ITS1 (5′-TCCGTAGGTGAACCTGCGG-3′) and ITS4 (5′-TCCTCCGCTTATTGATATGC-3′) (Al Halteet et al., 2020).

The PCR amplification was conducted in a 50-μl reaction mixture containing 10 μl 10 × PCR buffer, 5-μl templates, 1 μl ITS1 primer, 1 μl ITS4 primer, 0.5 μl Taq enzyme, 8 μl dNTP mixture, and 24.5 μl double-distilled water. The PCR amplification condition is as follows: 1 cycle of 95°C 10 min; 40 cycles of 95°C 30 s, 56°C 30 s, 72°C 30 s; 1 cycle of 72°C 10 min. The PCR products were sequenced with the same primers ITS1 and ITS4 (Sangon Biotech, China). The ITS sequences of the 32 yeast strains were identified by comparing the sequencing data with sequences in MycoBank using pairwise alignment of the MycoBank database^1^.

### The Morphology and Antifungal Susceptibility Analysis

The 32 yeast strains were inoculated on Sabouraud dextrose agar (SDA) with 0.01% chloramphenicol and chromogenic medium (CHROM agar), respectively. The plates were incubated at 28°C for 7 days, and the growth of the strains on the plates was assessed daily. The macroscopic characteristics and micromorphology of the strains on the plates were observed and determined. Meanwhile, the single colonies on the plates went through Gram staining and the microscopic characteristics were recorded. The Sensititre YeastOne YO10 (Thermo, Waltham, MA, United States) colorimetric panel was used for the antifungal susceptibility analysis of the 32 strains. The operation was performed according to the manufacturer’s instructions, and the interpretation for antifungal susceptibility testing was based on CLSI M59 and CLSI M60 (CLSI, 2020a,b).

### The MALDI-TOF MS Analysis

Pretreatment of strains: a single colony was picked in a 1.5-ml EP tube containing 1 ml 75% ethanol and 20–30 glass beads, mixed for 2 min. Then the EP tube was centrifuged at 12,000 rpm for 30 s. The supernatant was removed, and 70 μl freshly prepared 70% formic acid was added to the tube and mixed for 30 s. Then, 20 μl acetonitrile was added to the tube and mixed for 30 s. The tube was centrifuged at 12,000 rpm for 2 min. Two microliters of supernatant was added on the spot of the target plate, and 1 μl matrix was added to the same spot after the 2-μl supernatant dried. After the matrix dried, the target plate was taken to the mass spectrometer’s ionization chamber and the mass spectra of the strains were acquired using a VITEK MS Plus (bioMérieux, France) and the Launchpad v2.8 software program (Kratos, Manchester, United Kingdom). Then the cluster analysis of the mass spectra was carried out and dendrograms showing taxonomic relationships were prepared using the SARAMIS software (bioMérieux, France).

For instrument calibration, the *Escherichia coli* reference strain (ATCC 8739) was applied. The operation procedure was in line with the manufacturer’s instruction.

### Statistical Analysis

Kruskal–Wallis *H* test was used for the *S. ciferrii complex* species comparisons in the respective antifungal drugs. All results with *p* value < 0.05 were statistically significant.

## Results

### Identification of *Stephanoascus ciferrii* Complex Species

The occurrence of *S. ciferrii* complex in patients with CSOM was 53.13% (17/32) in male and 46.87% (15/32) in female. The age of the patients ranged from 20 to 70 with the median age of 36. The occurrence of *S. ciferrii* complex was 31.25% (10/32) in patients under 30 years old, 59.38% (19/32) in patients whose age were between 30 and 60 years old, and 9.37% (3/32) in patients above 60 years old. By ITS region alignment in the MycoBank, the 32 yeast strains of *S. ciferrii* complex were classified at species level. Based on the alignment results, there were 10 strains of *C. allociferrii* (31.25%), six strains of *C. mucifera* (18.75%), and 16 strains of *S. ciferrii* (50%) (Table 1). In our article, the reference strains for *S. ciferrii* involved CBS5295, CBS6546, and CBS6699. Although different strains of *S. ciferrii* had different corresponding reference strains (CBS5295, 6546, and 6699), the three reference strains all represented *S. ciferrii*.

**TABLE 1:** The identification of the 32 yeast strains of *S. ciferrii* complex isolated from CSOM patients using different methods.

**Strain number**	**Gender**	**Age (year)**	**MALDI-TOF MS**	**Sequencing of ITS region**		
				**Species**	**Similarity (%)**	**Reference strain**
S-1	F	30	*S. ciferrii*	*S. ciferrii*	99.61	CBS 5295
S-2	F	48	No result	*C. mucifera*	99.79	CBS 7409
S-3	M	36	No result	*C. allociferrii*	100.0	CBS 5166
S-4	F	28	No result	*C. allociferrii*	98.90	CBS 5166
S-5	M	27	No result	*C. allociferrii*	99.63	CBS 5166
S-6	M	49	*S. ciferrii*	*S. ciferrii*	100.0	CBS 5295
S-7	M	31	*S. ciferrii*	*S. ciferrii*	99.61	CBS 5295
S-8	F	28	No result	*C. allociferrii*	98.90	CBS 5166
S-9	M	32	No result	*C. mucifera*	99.66	CBS 7409
S-10	M	23	*S. ciferrii*	*S. ciferrii*	99.01	CBS 5295
S-11	M	43	*S. ciferrii*	*S. ciferrii*	99.61	CBS 5295
S-12	M	62	No result	*C. mucifera*	99.66	CBS 7409
S-13	F	52	No result	*C. allociferrii*	99.63	CBS 5166
S-14	M	70	*S. ciferrii*	*S. ciferrii*	99.61	CBS 5295
S-15	F	49	*S. ciferrii*	*S. ciferrii*	100.0	CBS 5295
S-16	F	56	No result	*C. allociferrii*	100.0	CBS 5166
S-17	F	32	No result	*C. allociferrii*	99.08	CBS 5166
S-18	M	27	*S. ciferrii*	*S. ciferrii*	99.61	CBS 5295
S-19	M	31	*S. ciferrii*	*S. ciferrii*	99.61	CBS 5646
S-20	F	37	*S. ciferrii*	*S. ciferrii*	99.61	CBS 5295
S-21	F	54	No result	*C. allociferrii*	100.0	CBS 5166
S-22	M	42	*S. ciferrii*	*S. ciferrii*	100.0	CBS 6699
S-23	M	35	No result	*C. mucifera*	99.79	CBS 7409
S-24	F	66	No result	*C. allociferrii*	99.63	CBS 5166
S-25	M	40	No result	*C. allociferrii*	98.88	CBS 5166
S-26	F	36	*S. ciferrii*	*S. ciferrii*	99.58	CBS 5646
S-27	F	46	No result	*C. mucifera*	98.42	CBS7409
S-28	F	45	*S. ciferrii*	*S. ciferrii*	100.0	CBS 5295
S-29	F	29	*S. ciferrii*	*S. ciferrii*	100.0	CBS 5646
S-30	M	20	*S. ciferrii*	*S. ciferrii*	99.61	CBS 5295
S-31	M	26	*S. ciferrii*	*S. ciferrii*	99.60	CBS 5295
S-32	M	29	No result	*C. mucifera*	99.58	CBS7409

*M, male; F, female; *S. ciferrii*, *Stephanoascus ciferrii*; *C. mucifera*, *Candida mucifera*; *C. allociferrii*, *Candida allociferrii*; CBS, Centraalbureau voor Schimmelcultures, Delft, Netherlands.*

### The Morphology and Antifungal Susceptibility Analysis

**FIGURE 1:**
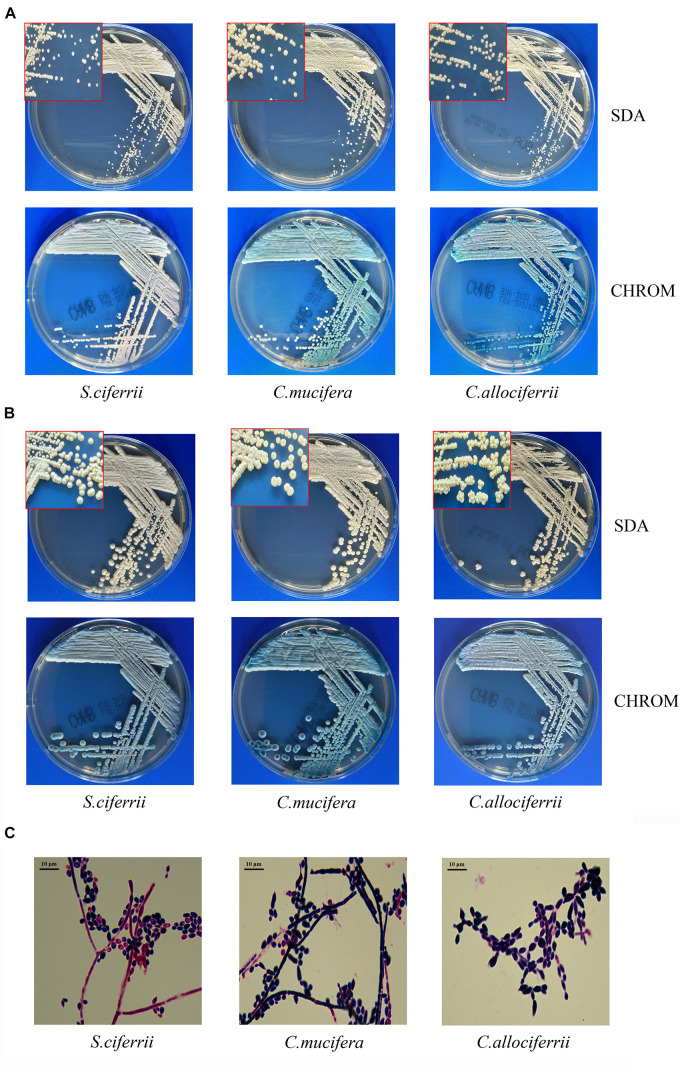
The morphological characteristics of *S. ciferrii* complex species. **(A)** Colonies of *S. ciferrii* complex species cultured on an SDA plate and CHROM agar at 28°C for 72 h. **(B)** Colonies of *S. ciferrii* complex species cultured on an SDA plate and CHROM agar at 28°C for 7 days. **(C)** The Gram staining of colonies of *S. ciferrii* complex species cultured at 28°C for 72 h, × 1,000.

The morphology of the three species cultured on the SDA plate and CHROM agar plate at 28°C for 72 h and 7 days is shown in Figures 1A,B, respectively. There were small, round, smooth, and milky white colonies that could be seen after 48 h of culture on the SDA plate. After 72 h, the colonies embedded to the agar and the texture became hard. It was not easy to scrape or grind the colonies from the agar. A week later, creamy or slightly yellowish colonies could be seen on the SDA plate and the center of the colonies was gyrus-like or cauliflower-like. Besides, the colonies of some *C. mucifera* isolates became sunken. On the CHROM agar plate, the colonies also grew well and they were inlaid into plates 72 h later. The colonies were regular round with a blue center and white edge. The colonies became rough with gyrus-like grooves and irregular edges 7 days later. The Gram staining of the three species is shown in Figure 1C. Under the microscope, there were gram-positive yeast-like fungi and slender hyphae. The spores were single-celled and ovoid, distributing along the periphery of the hyphae. The images about all the 32 isolated species’ appearance on SDA and CHROM agar at 28°C for 3 and 7 days were provided in the Supplementary Material, as well as the Gram staining pictures. Actually, the macroscopic and microscopic characteristics of the three species were similar, making it hard to distinguish them based on morphology.

**FIGURE 2:**
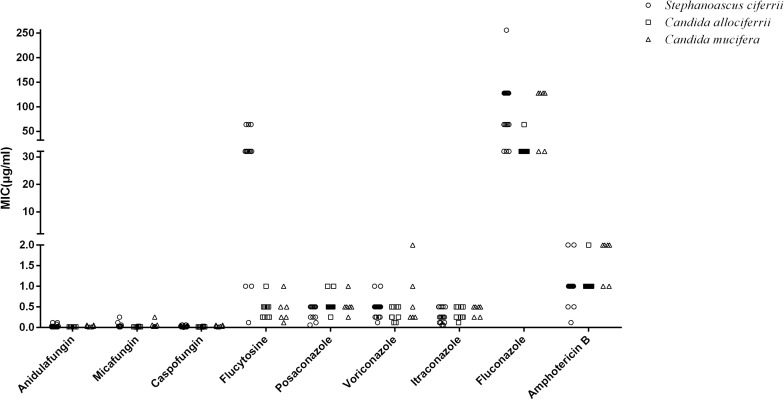
The distribution of the MIC values of *S. ciferrii* complex species for different antifungal drugs.

The MICs of the 32 isolated strains in the antifungal susceptibility testing are shown in Table 2, and the distribution is shown in Figure 2. The MICs for anidulafungin, micafungin, and caspofungin were relatively small. The MICs for anidulafungin, micafungin, and caspofungin ranged between 0.015 and 0.12 μg/ml, 0.008 and 0.25 μg/ml, and 0.006 and 0.06 μg/ml, respectively. There was statistical difference between the MICs of *C. allociferrii* and those of *C. mucifera* for anidulafungin, micafungin, and caspofungin (*p* < 0.05), indicating that *C. mucifera* tended to have a higher MIC value compared with that of *C. allociferrii* for the echinocandins. However, the antifungal susceptibility of the 32 strains for azole antifungal drugs differed. The MICs for posaconazole, voriconazole, itraconazole, and fluconazole ranged between 0.06 and 1 μg/ml, 0.12 and 2 μg/ml, 0.06 and 0.5 μg/ml, and 32 and 256 μg/ml, respectively. As for posaconazole, voriconazole, and itraconazole, the 32 strains had relatively low MICs compared with fluconazole and there were no differences among the antifungal susceptibility of the three species for the three antifungal drugs (*p* < 0.05). For fluconazole, the three species of *S. ciferrii* complex owned their characteristics. The MICs of *C. allociferrii* for fluconazole ranged between 32 and 64 μg/ml. Nine of the 10 *C. allociferrii* strains (90%) had the MICs of 32 μg/ml. The MICs of *C. mucifera* for fluconazole ranged between 32 and 128 μg/ml, and four of the six *C. mucifera* strains (66.67%) had MICs of 128 μg/ml. As for *S. ciferrii*, 50% (8/16) strains had MICs equal or greater than 128 μg/ml and 31.25% (5/16) strains had MICs of 64 μg/ml. The *S. ciferrii* and *C. mucifera* tended to have high fluconazole MICs compared with *C. allociferrii* (*p* < 0.05). For flucytosine, the MICs of *C. allociferrii* and *C. mucifera* ranged from 0.25 to 1 μg/ml and 0.25 to 2 μg/ml, respectively. However, 81.25% (13/16) of the *S. ciferrii* had MICs of 32 or 64 μg/ml. *S. ciferrii* tended to have higher MICs for flucytosine compared with *C. allociferrii* and *C. mucifera* (*p* < 0.05). As for amphotericin B, the MICs ranged between 0.12 and 2 μg/ml and there was a difference between the MICs of *S. ciferrii* and those of *C. mucifera* (*p* < 0.05). *C. mucifera* tended to have a higher MIC value compared with that of *S. ciferrii* for amphotericin B.

**TABLE 2:** The MICs of the 32 isolated strains in the antifungal susceptibility testing with microbroth dilution method.

**Strain number**	**Species**	**MIC values (μ g/mL)**								
		**Anidulafungin**	**Micafungin**	**Caspofungin**	**Flucytosine**	**Posaconazole**	**Voriconazole**	**Itraconazole**	**Fluconazole**	**Amphotericin B**
S-1	*S. ciferrii*	0.015	0.015	0.015	32	0.5	0.5	0.5	128	0.12
S-2	*C. mucifera*	0.06	0.06	0.03	0.5	0.25	0.25	0.25	32	2
S-3	*C. allociferrii*	0.015	0.015	0.008	0.25	0.5	0.12	0.25	32	1
S-4	*C. allociferrii*	0.015	0.03	0.03	1	0.5	0.5	0.5	32	1
S-5	*C. allociferrii*	0.015	0.015	0.015	0.25	0.25	0.25	0.12	32	1
S-6	*S. ciferrii*	0.08	0.25	0.06	1	0.5	0.5	0.5	256	2
S-7	*S. ciferrii*	0.015	0.015	0.015	32	0.5	0.25	0.25	32	1
S-8	*C. allociferrii*	0.015	0.015	0.015	0.5	1	0.5	0.5	64	1
S-9	*C. mucifera*	0.015	0.25	0.06	0.5	0.5	1	0.5	128	2
S-10	*S. ciferrii*	0.015	0.12	0.03	64	0.5	0.5	0.25	64	1
S-11	*S. ciferrii*	0.015	0.015	0.015	32	0.5	0.5	0.5	128	1
S-12	*C. mucifera*	0.03	0.03	0.015	0.12	0.5	2	0.5	128	1
S-13	*C. allociferrii*	0.015	0.015	0.008	0.25	0.5	0.25	0.25	32	2
S-14	*S. ciferrii*	0.03	0.03	0.015	64	0.5	0.5	0.25	128	1
S-15	*S. ciferrii*	0.12	0.06	0.06	1	0.5	1	0.5	128	1
S-16	*C. allociferrii*	0.015	0.015	0.015	0.5	0.5	0.25	0.5	32	1
S-17	*C. allociferrii*	0.015	0.015	0.008	0.5	0.5	0.5	0.25	32	1
S-18	*S. ciferrii*	0.03	0.03	0.006	32	0.5	1	0.5	128	1
S-19	*S. ciferrii*	0.015	0.015	0.03	32	0.06	0.25	0.06	64	0.5
S-20	*S. ciferrii*	0.03	0.03	0.06	32	0.25	0.5	0.12	128	0.5
S-21	*C. allociferrii*	0.015	0.015	0.015	0.5	0.5	0.12	0.25	32	1
S-22	*S. ciferrii*	0.12	0.03	0.06	32	0.12	0.12	0.06	32	1
S-23	*C. mucifera*	0.06	0.06	0.06	0.25	1	0.5	0.5	128	1
S-24	*C. allociferrii*	0.015	0.008	0.015	0.25	1	0.5	0.5	32	1
S-25	*C. allociferrii*	0.015	0.015	0.015	0.5	0.5	0.25	0.25	32	1
S-26	*S. ciferrii*	0.03	0.03	0.03	64	0.5	0.5	0.5	128	2
S-27	*C. mucifera*	0.03	0.03	0.03	1	0.5	0.25	0.25	128	2
S-28	*S. ciferrii*	0.015	0.03	0.015	0.12	0.5	0.5	0.25	64	1
S-29	*S. ciferrii*	0.015	0.015	0.015	32	0.25	0.25	0.12	64	1
S-30	*S. ciferrii*	0.015	0.015	0.015	32	0.25	0.25	0.12	32	1
S-31	*S. ciferrii*	0.015	0.03	0.03	32	0.5	0.5	0.25	64	1
S-32	*C. mucifera*	0.015	0.03	0.03	0.25	0.5	0.25	0.5	32	2

### The MALDI-TOF MS Identification and Cluster Analysis of the Mass Spectra

**FIGURE 3:**
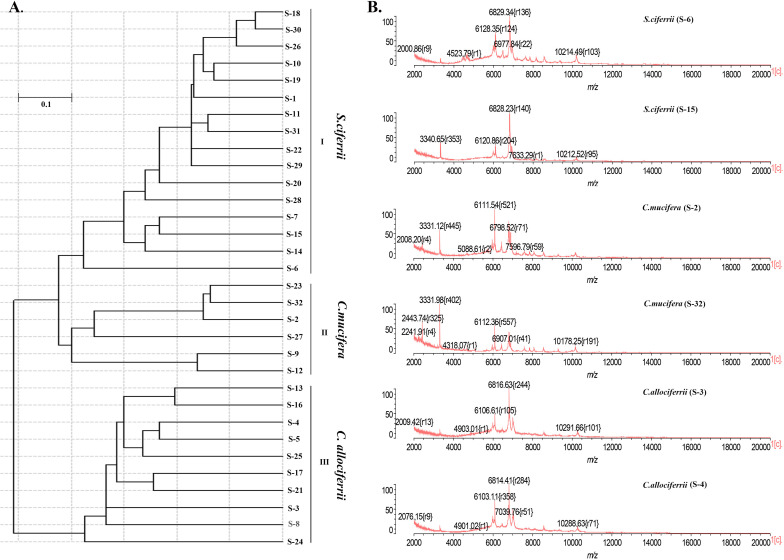
The classification of the 32 isolated *S. ciferrii* complex strains at species level. **(A)** The clustering dendrogram of the 32 *S. ciferrii* complex isolates created by SARAMIS. **(B)** The mass spectra of six *S. ciferrii* complex strains.

Apart from identification by ITS sequencing, the 32 isolated strains all went through identification by MALDI-TOF MS. The 16 strains of *S. ciferrii* were identified as *S. ciferrii* with 99.9% similarity by MALDI-TOF MS. However, MALDI-TOF MS was unable to identify the 10 strains of *C. allociferrii* and six strains of *C. mucifera*, showing no results (Table 1). For MALDI-TOF MS, the limitation lies in its inability to identify non-clinically validated species or species not included in the MALDI-TOF database (Mesureur et al., 2018). The database of MALDI-TOF MS did not involve the reference database of *C. allociferrii* and *C. mucifera.* Thus, the MALDI-TOF MS could not identify the *S. ciferrii* complex at species level. The cluster analysis of the mass spectra of the 32 isolated strains is shown in Figure 3A. By cluster analysis with SARAMIS, the 32 isolated strains were divided into three clusters. All the *S. ciferrii* strains were divided into cluster I, all the *C. mucifera* strains belonged to cluster II, and all the *C. allociferrii* strains were assigned to cluster III. The mass spectra of two isolates from each species are shown in Figure 3B. The three species of *S. ciferrii* complex had their own special peaks in the mass spectra, making it possible to identify those using MALDI-TOF MS by constructing reference databases at species level. The mass spectra of all the 32 strains were provided in the Supplementary Material.

## Discussion

*Stephanoascus ciferrii*, which is first discovered in 1965, is an opportunistic rare yeast pathogen with low prevalence (<1% of clinical *Candida* infections) (Kreger-Van Rij, 1965; Mixao et al., 2019). The literature about *S. ciferrii* is limited, and it is usually reported in some sporadic cases of infections (Villanueva-Lozano et al., 2016). Reports on *S. ciferrii* complex are even more limited. Hironobu Soki reported a rare case of intraorbital abscess caused by *C. allociferrii*, which is the only literature describing *S. ciferrii* complex since the *S. ciferrii* complex was proposed (Soki et al., 2015). The clinical isolation rate of *S. ciferrii* complex is low. Moreover, in the majority of cases, species of the *S. ciferrii* complex are not identified. CSOM is prevalent in China just like most other developing countries and usually caused by bacteria (Xu et al., 2020). CSOM caused by *S. ciferrii* complex is rarely reported. In our study, we had collected 32 strains of *S. ciferrii* complex isolated from patients with CSOM, indicating that more attention should be paid to these species. In our study, the *S. ciferrii* complex could occur in young, middle-aged, and elderly people. However, the occurrence of *S. ciferrii* complex mainly focused on the patients whose ages were between 30 and 60 years and showed little relationship with gender. Since the number of clinical isolates at the species level was too small, we did not calculate the occurrence of *S. ciferrii* complex at species level. Although it is hard to find a firm association from the limited number of clinical isolates in our study, the small sample number is relative and could still be highly informative due to the rare frequency of these species.

By sequencing of the ITS region, the 32 strains were identified as *S. ciferrii*, *C. allociferrii*, and *C. mucifera.* The morphology analysis of the three species shows us the basic phenotypic characteristics of *S. ciferrii* complex species. The morphology characteristics of the three species are similar, indicating that it is hard to distinguish them based on macroscopic and microscopic characteristics. As for the antifungal susceptibility profiles of *S. ciferrii* complex, the three species all had low MICs for echinocandins, making this kind of antifungal a good choice to treat *S. ciferrii* complex infection. As the MICs of *S. ciferrii* complex species for echinocandins and amphotericin B were relatively low, the MIC differences among the *S. ciferrii* complex species do not have big effects on the clinical medication. As for the azole antifungal drugs, the MICs for posaconazole, voriconazole, and itraconazole were relatively much smaller than MICs for fluconazole. However, for fluconazole, all the strains had high MICs, which is consistent with the research by Antonio Pérez-Hansen (Perez-Hansen et al., 2019). In the research by Antonio Pérez-Hansen on antifungal susceptibility profiles of rare ascomycetous yeasts, *S. ciferrii* complex was found to have high fluconazole MICs (>32 μg/ml). Moreover, the antifungal susceptibility to fluconazole at species level revealed that the *S. ciferrii* and *C. mucifera* tended to have high fluconazole MICs compared with *C. allociferrii*. Fluconazole is a triazole antifungal that inhibits fungal cytochrome P450 activity, decreasing ergosterol synthesis and inhibiting cell membrane formation (Hornik et al., 2021). Sasani et al. (2021) reported that the formation of biofilms and the overexpression of *ERG11* and *UPC2* genes in *Candida tropicalis* were related with fluconazole resistance. Abbes et al. (2021) found that *Mdr* gene overexpression in *Trichosporon asahii* isolates was significantly associated with fluconazole resistance. However, there are no articles reporting why fluconazole resistance is high also in this complex. Perhaps, the fluconazole resistance in *S. ciferrii* complex is related with the formation of biofilms and the overexpression of some certain genes. In the future study, we plan to explore the mechanism of fluconazole resistance *S. ciferrii* complex to verify these assumptions. Moreover, the antifungal susceptibility specificity at species level became more obvious for flucytosine. All the strains of *C. mucifera* and *C. allociferrii* had relatively low MICs while most of the *S. ciferrii* owned high flucytosine MICs. However, there were three strains of *S. ciferrii* with low MICs (≤1 μg/ml). The phenomenon may be worth our attention, and further study to explore the resistance discrepancy is needed.

MALDI-TOF MS, as a rapid, reliable, and high-throughput diagnostic tool, has entered clinical microbiology diagnostics and is wildly used for fungal identification (Delavy et al., 2019). Although ITS region sequencing could identify clinically important yeasts accurately, it is costly and time consuming (Ciardo et al., 2006; Hou et al., 2016). In our study, the strains of *C. mucifera* and *C. allociferrii* could not be identified by MALDI-TOF MS while the cluster analysis of the mass spectra divided the 32 strains into three clusters. The three clusters represented the three species of the *S. ciferrii* complex, suggesting that it is possible to identify *S. ciferrii* complex species using MALDI-TOF MS. The lack of reference database for *C. allociferrii* and *C. mucifera* limits the ability of MALDI-TOF MS in identification, making it essential to construct the reference database of *S. ciferrii* complex species. Taverna et al. (2019) developed an in-house database of 143 strains belonging to 42 yeast species in the MALDI Biotyper platform, making it possible to differentiate closely related species and distinguish the molecular genotypes of *Cryptococcus neoformans* and *Cryptococcus gattii*. Moreover, Veloo et al. (2018) optimized the identification of clinical anaerobic isolates with MALDI-TOF MS using 6,309 anaerobic isolates, thus increasing the number of anaerobic isolates that could be identified with high confidence. Therefore, it is recommended to expand or construct the databases that include less frequently isolated species to identify them quickly and accurately by MALDI-TOF MS, gaining valuable insight into the clinical relevance of the rare microorganism.

In summary, 32 strains of *S. ciferrii* complex isolated from patients with CSOM were identified at species level by the sequencing of ITS regions. The morphology of the three species was observed in our study in detail. The antifungal susceptibility profiles of three species of *S. ciferrii* complex exhibited differences with each other. *S. ciferrii* and *C. mucifera* tended to have high fluconazole MICs compared with *C. allociferrii*. *C. mucifera* and *C. allociferrii* had relatively low flucytosine MICs while *S. ciferrii* owned high flucytosine MICs. Besides, *C. mucifera* tended to have a higher MIC value than *S. ciferrii* for amphotericin B and *C. allociferrii* for anidulafungin, micafungin, and caspofungin. Apart from the sequencing of ITS regions, MALDI-TOF MS was used to identify *S. ciferrii* complex at species level. The MALDI-TOF MS showed no results to *C. mucifera* and *C. allociferrii*. Since the cluster analysis of the mass spectra could distinguish the three groups, construction of a reference database of *C. mucifera* and *C. allociferrii* is required for subsequent species identification. However, the number of *S. ciferrii* complex strains in our study is limited and more strains are required to perfect the morphology characteristics of this complex and its antifungal susceptibility profiles. Besides, more mass spectra of *C. mucifera* and *C. allociferrii* are needed to construct the reference database, enabling the MALDI-TOF MS to identify *S. ciferrii* complex at species level.

## Data Availability Statement

The original contributions presented in the study are included in the article/Supplementary Material, further inquiries can be directed to the corresponding author/s.

## Author Contributions

PG, KL, and YP participated in research design and data analysis. ZW participated in the performance of the research and data analysis. PL participated in the performance of the research. YC contributed new reagents or analytic tools. YH participated in the writing of the manuscript and data analysis. All authors contributed to the article and approved the submitted version.

## Conflict of Interest

The authors declare that the research was conducted in the absence of any commercial or financial relationships that could be construed as a potential conflict of interest.

## Footnotes

1 https://www.mycobank.org/page/Pairwise_alignment
