# Supplementary material for: Identification and Antifungal Susceptibility Analysis of Stephanoascus ciferrii Complex Species Isolated From Patients With Chronic Suppurative Otitis Media
Source: Front Microbiol. 2021 Jul 21;12:680060. doi: 10.3389/fmicb.2021.680060 (PMC8334361; doi:10.3389/fmicb.2021.680060)
Supplement: Supplementary file 1 [file Data_Sheet_1.pdf]

S-1

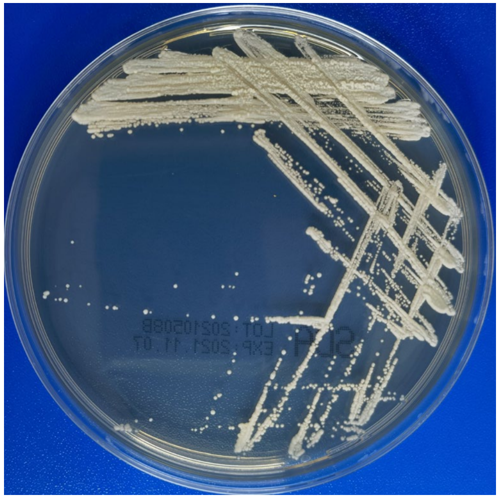

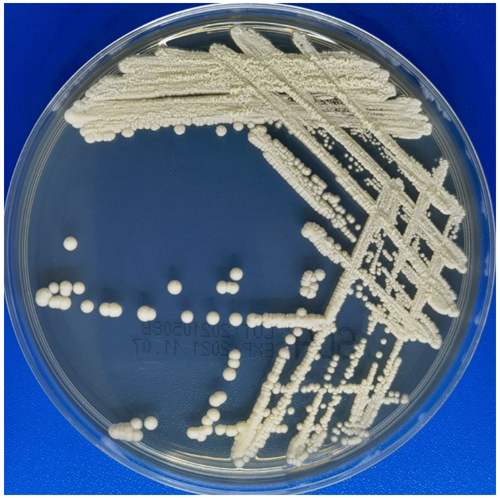

SDA, 28°C,72h

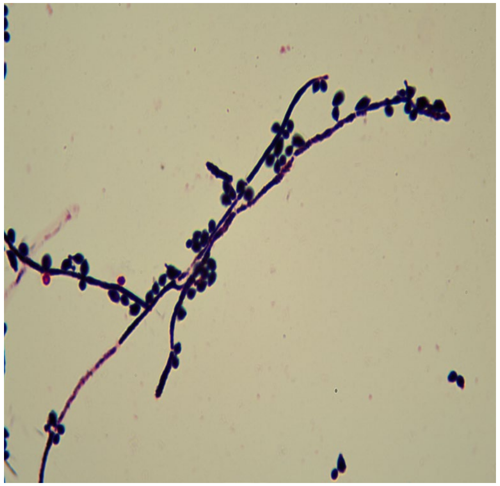

Gram staining

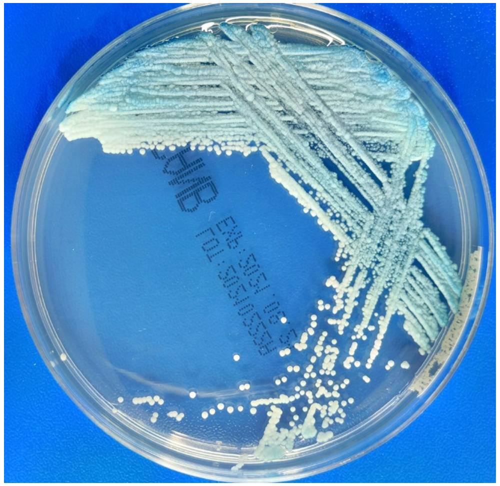

CHROM, 28°C, 72h

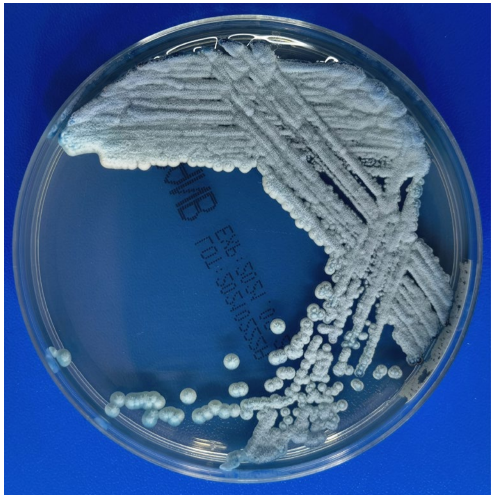

CHROM, 28°C,7d

S-2

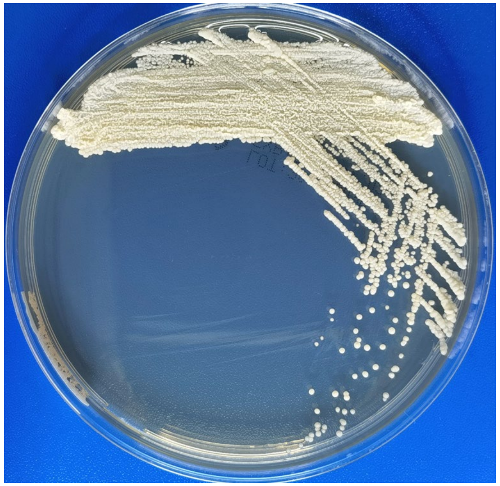

SDA, 28°C,72h

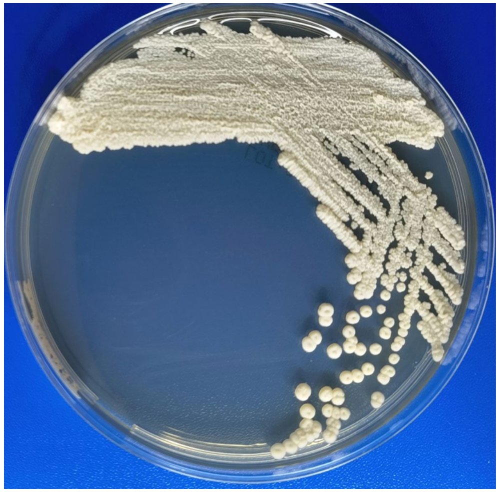

SDA, 28°C,7d

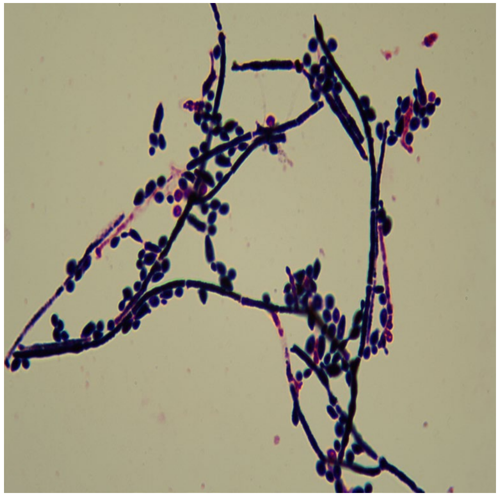

Gram staining

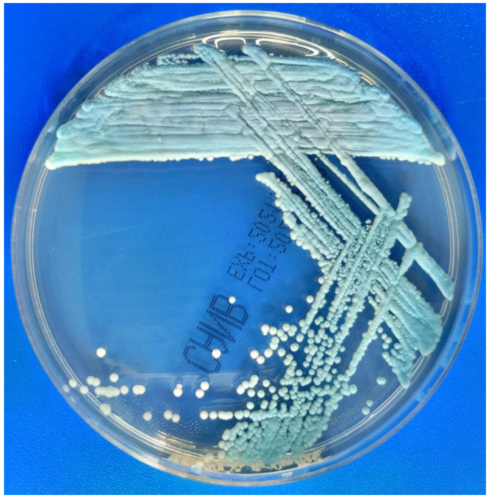

CHROM, 28°C, 72h

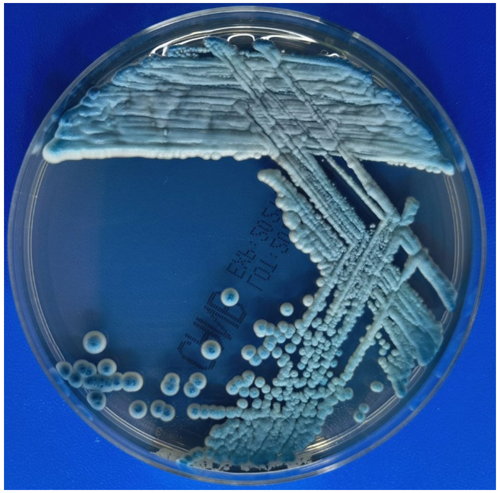

CHROM, 28°C,7d

S-3

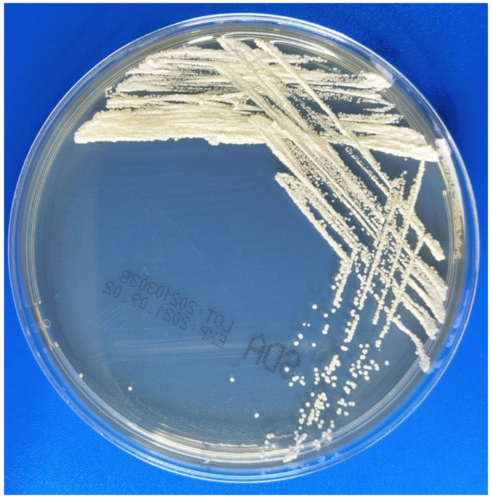

SDA, 28°C,72h

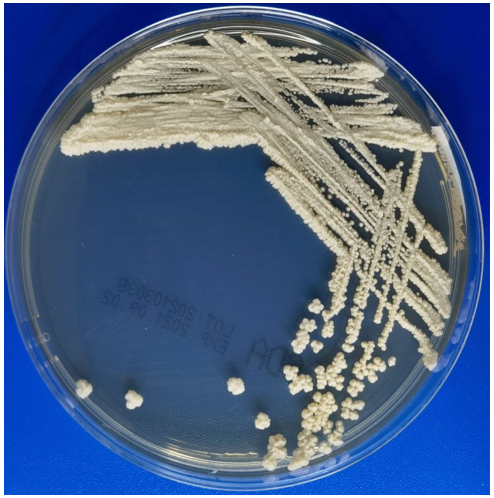

SDA, 28°C,7d

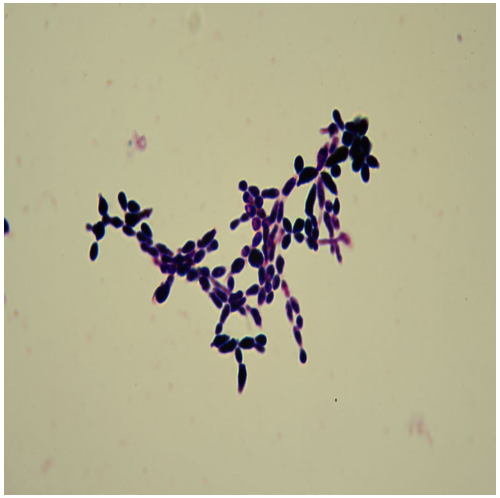

Gram staining

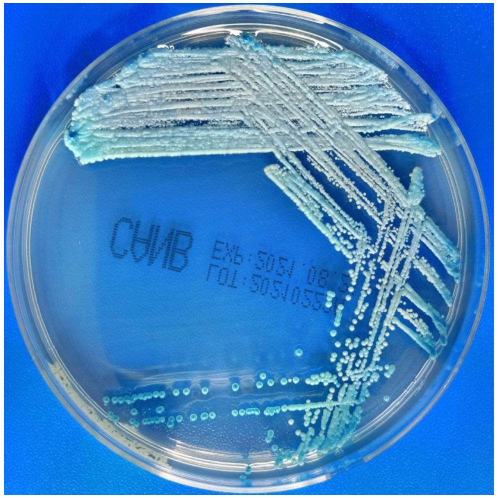

CHROM, 28°C, 72h

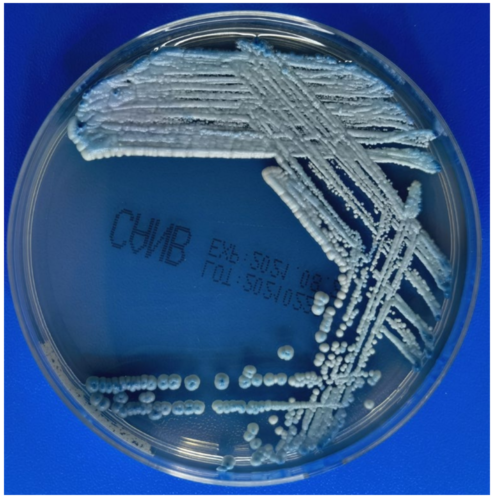

CHROM, 28°C,7d

S-4

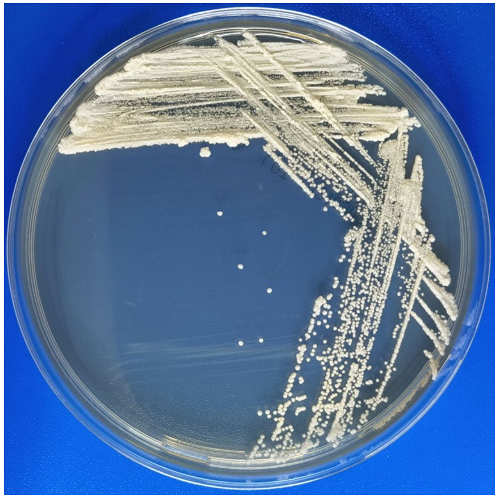

SDA, 28°C,72h

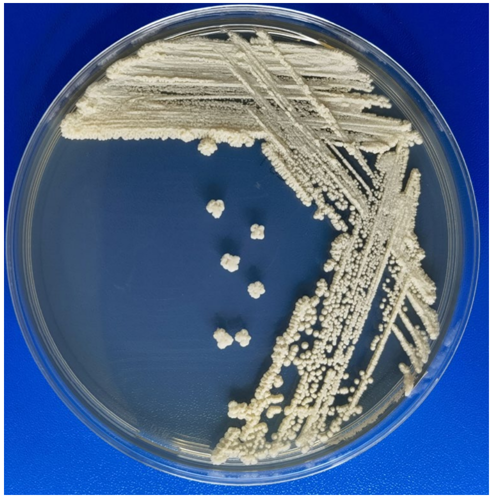

SDA, 28°C,7d

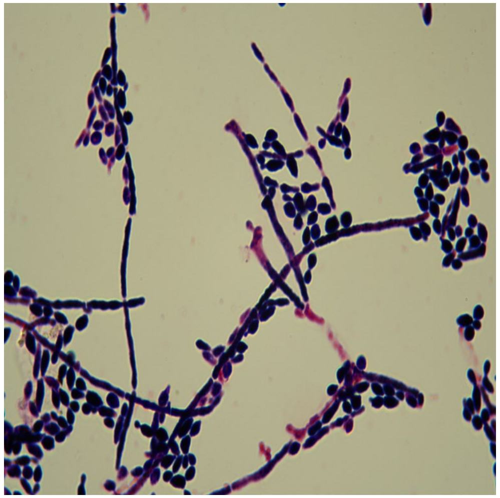

Gram staining

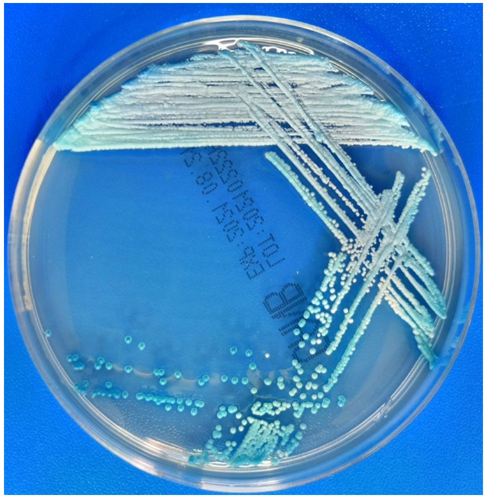

CHROM, 28°C, 72h

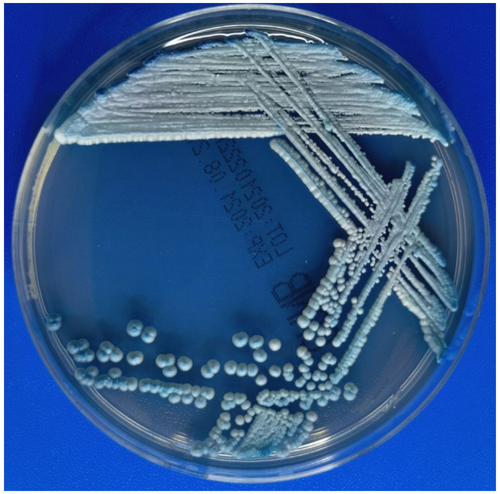

CHROM, 28°C,7d

S-5

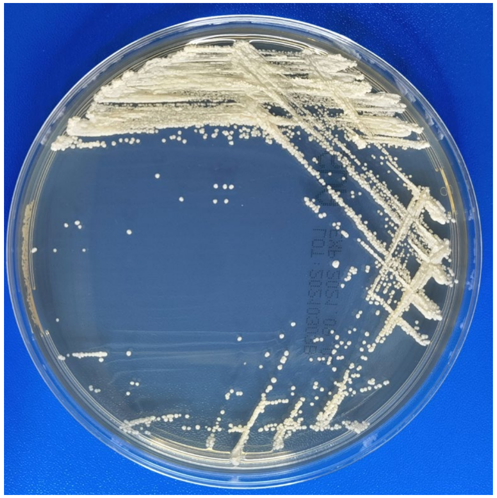

SDA, 28°C,72h

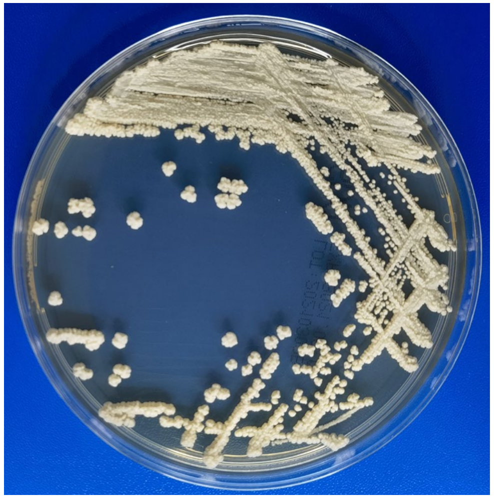

SDA, 28°C,7d

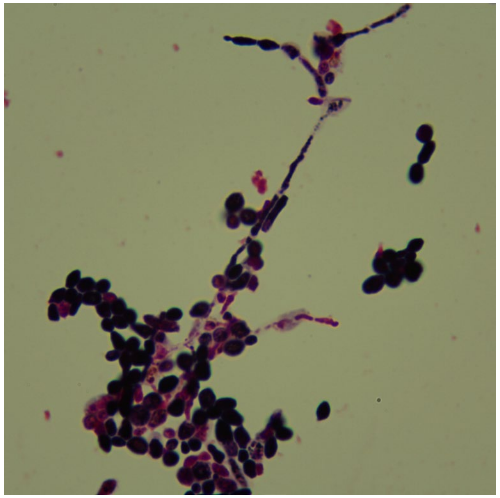

Gram staining

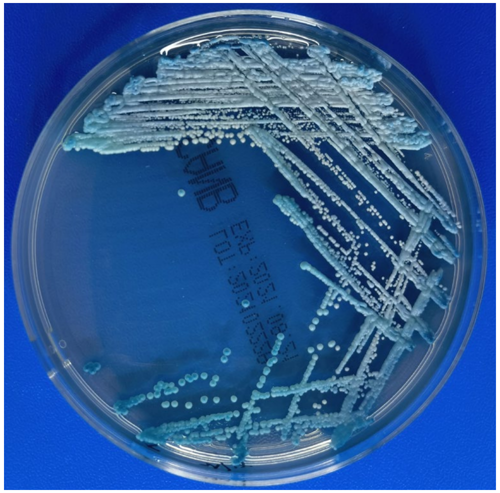

CHROM, 28°C, 72h

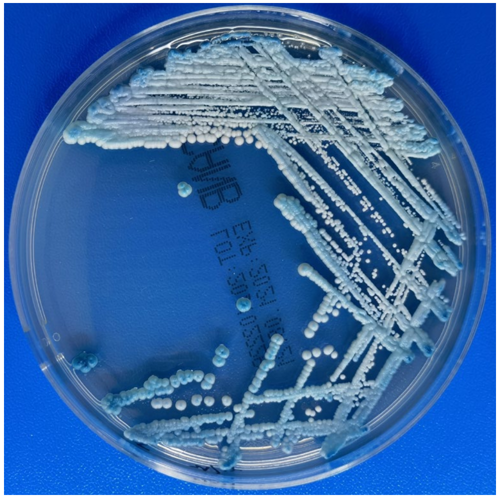

CHROM, 28°C,7d

S-6

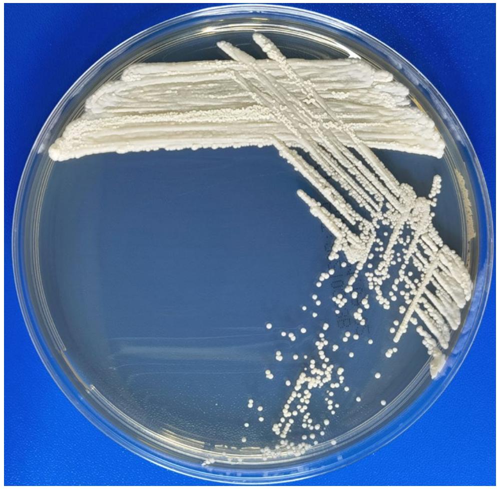

SDA, 28℃,72h

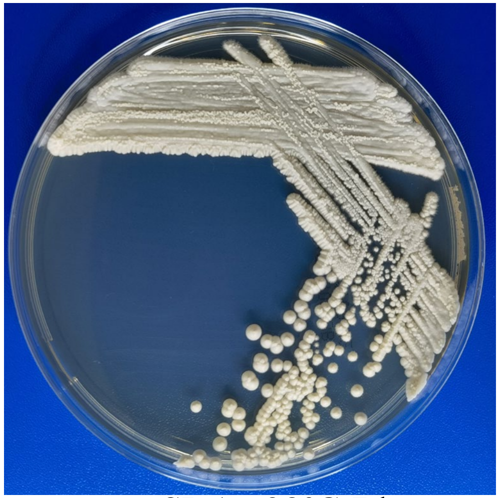

SDA, 28℃,7d

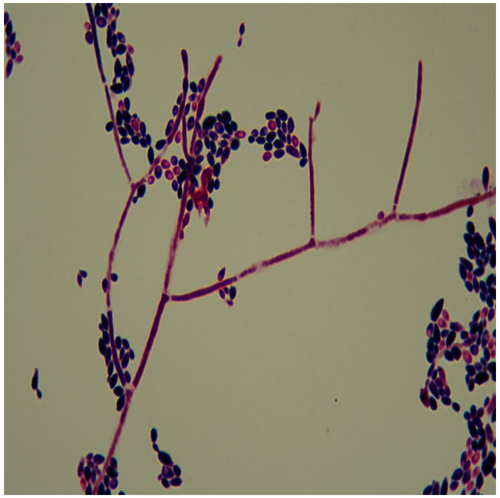

Gram staining

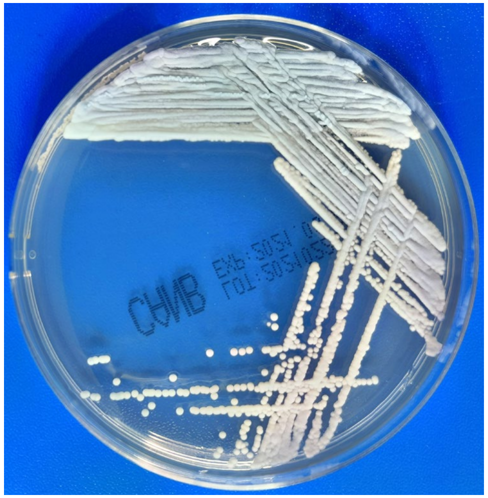

CHROM, 28℃, 72h

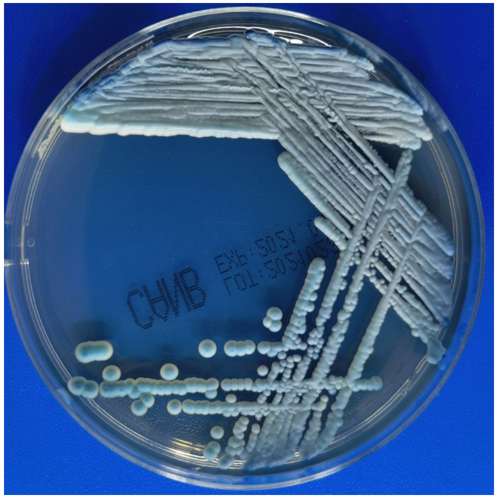

CHROM, 28℃,7d

S-7

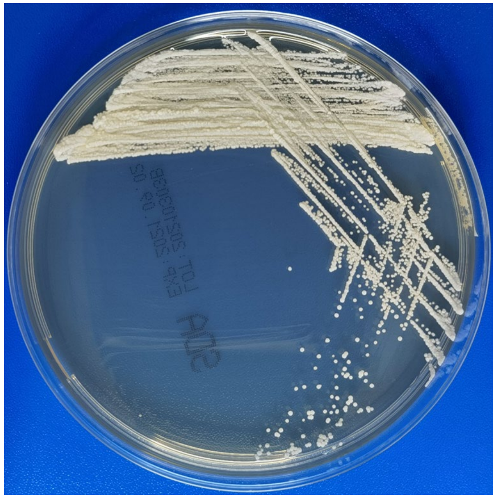

SDA，28℃,72h

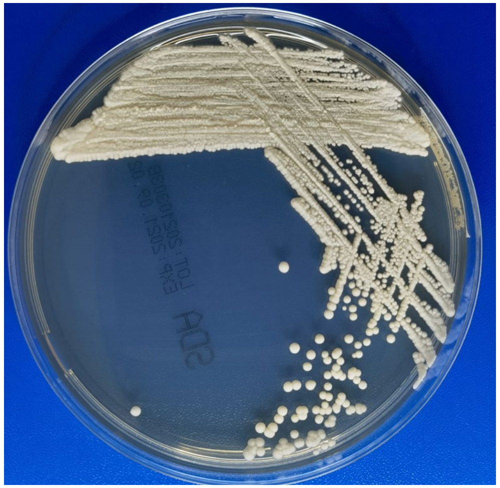

SDA，28℃,7d

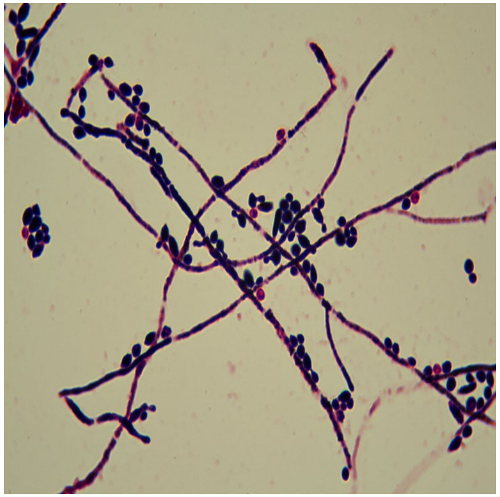

Gram staining

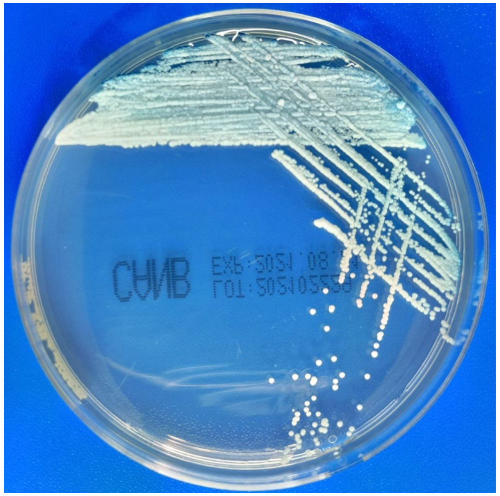

CHROM，28℃, 72h

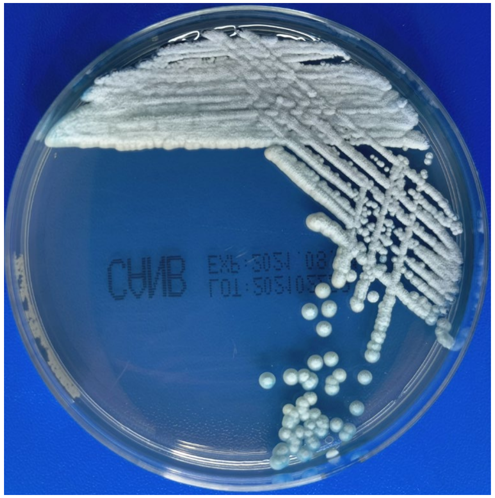

CHROM，28℃,7d

S-8

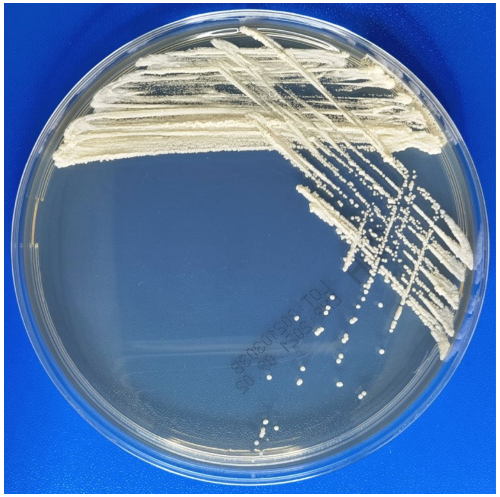

SDA, 28°C,72h

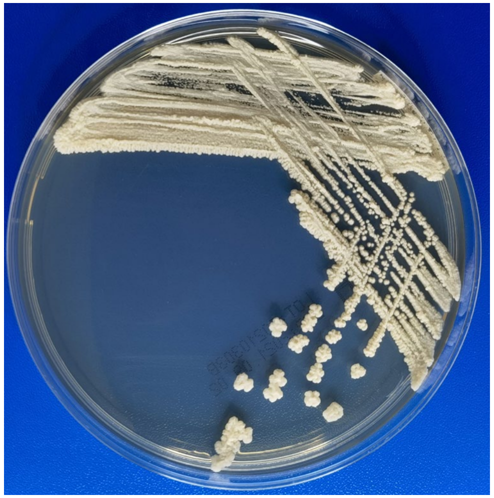

SDA, 28°C,7d

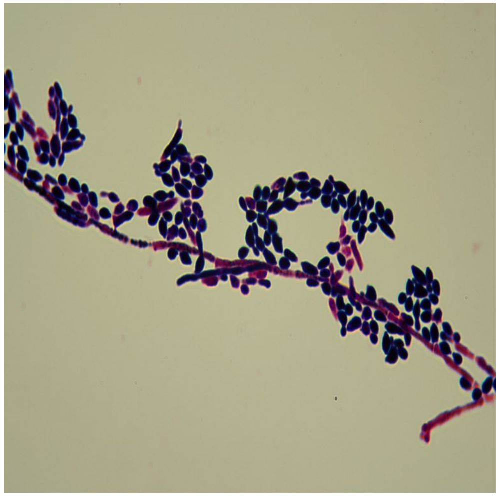

Gram staining

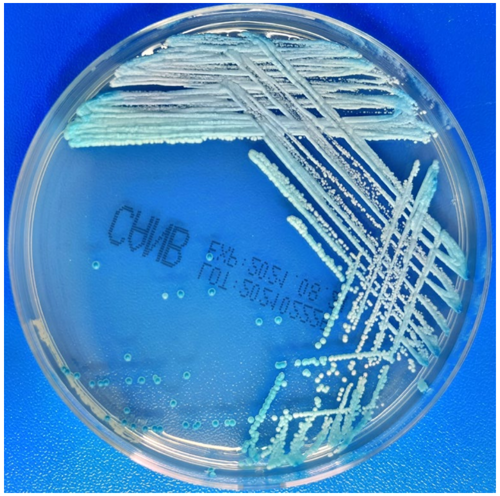

CHROM, 28°C, 72h

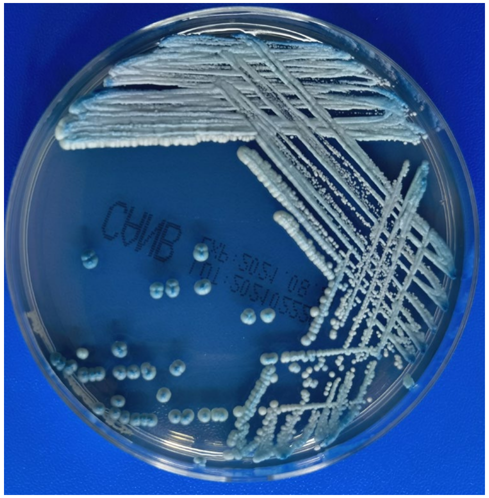

CHROM, 28°C,7d

S-9

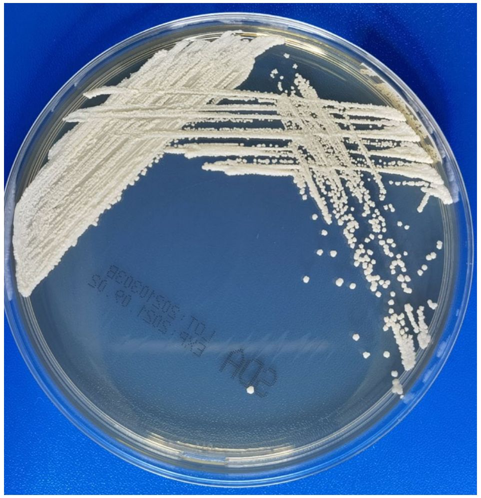

SDA, 28℃,72h

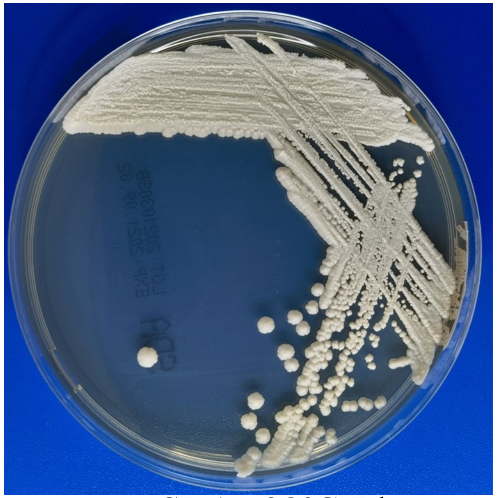

SDA, 28℃,7d

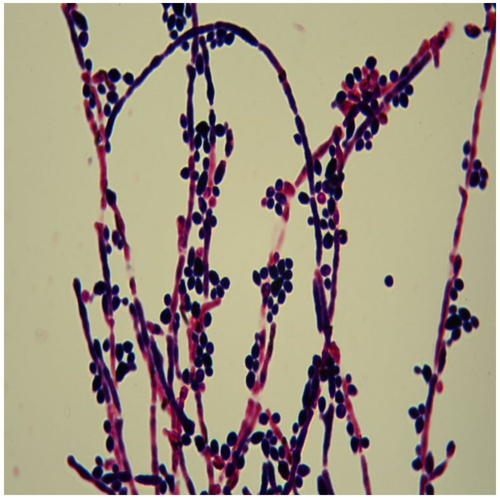

Gram staining

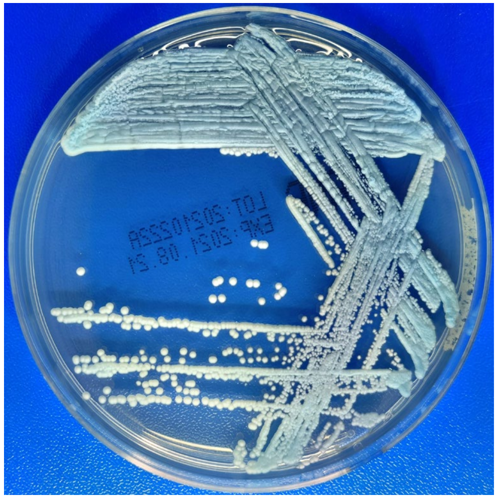

CHROM, 28℃, 72h

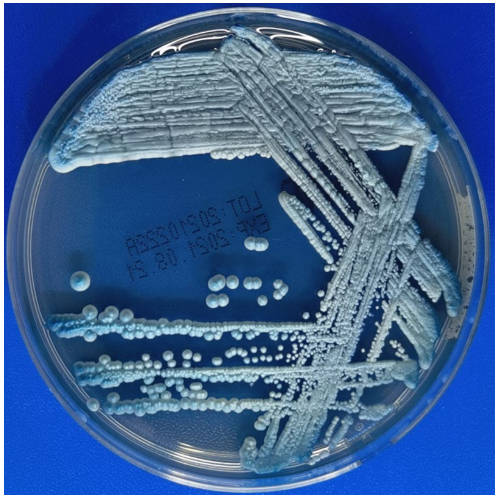

CHROM, 28℃,7d

S-10

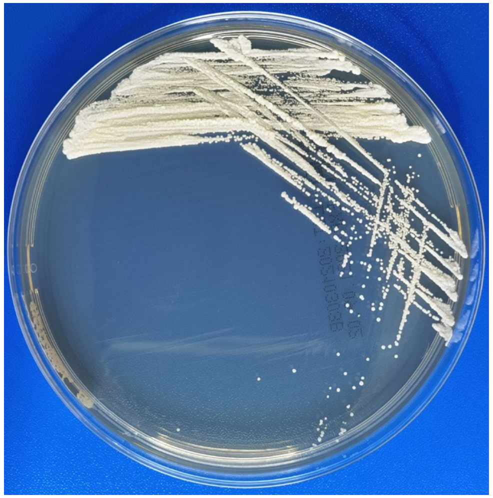

SDA，28℃,72h

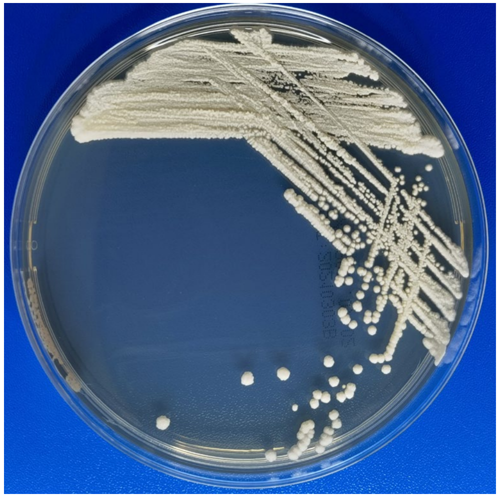

SDA，28℃,7d

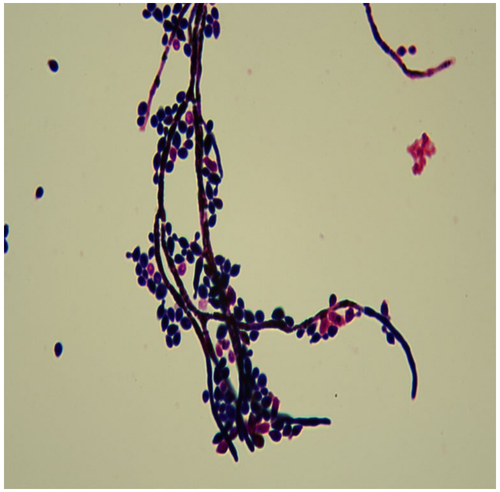

Gram staining

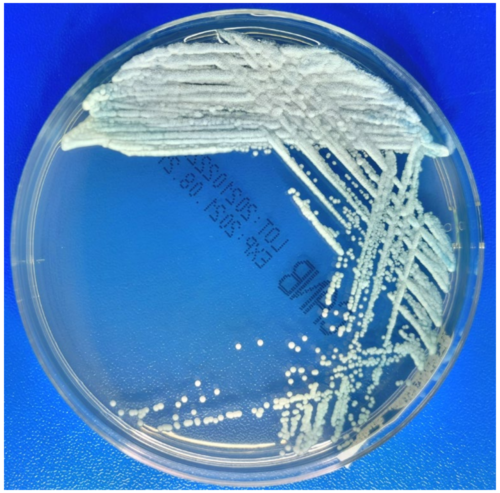

CHROM，28℃, 72h

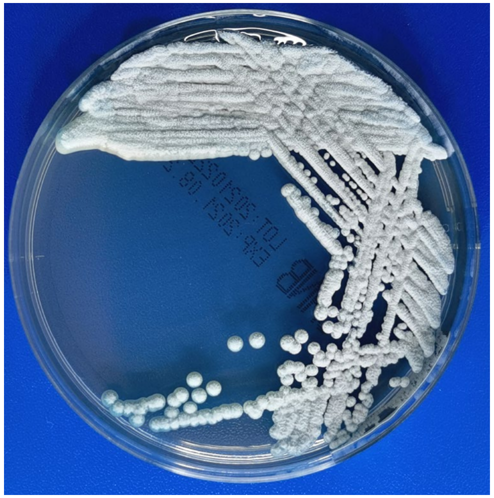

CHROM，28℃,7d

S-11

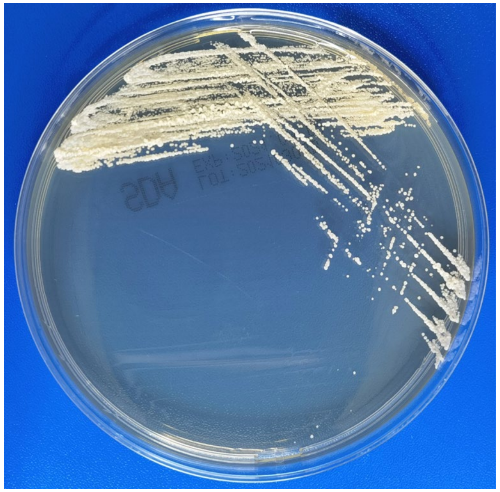

SDA, 28°C,72h

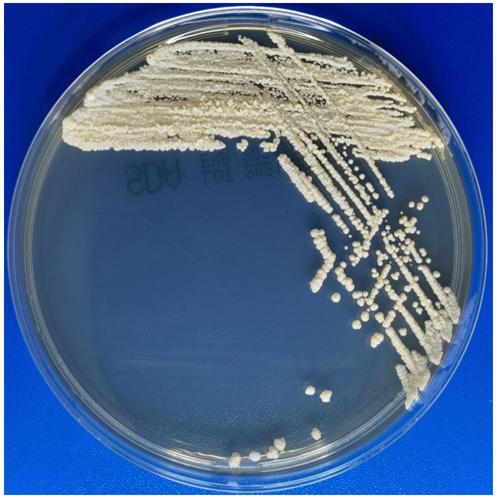

SDA, 28°C,7d

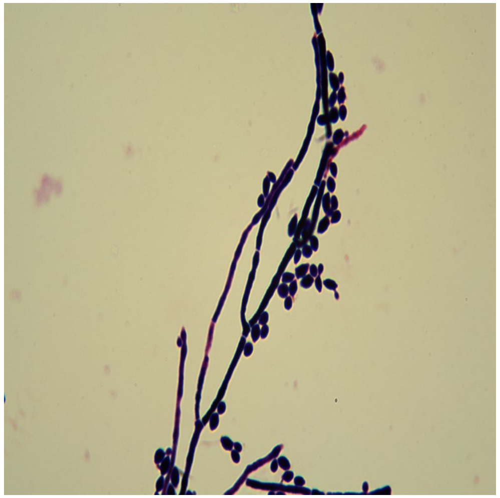

Gram staining

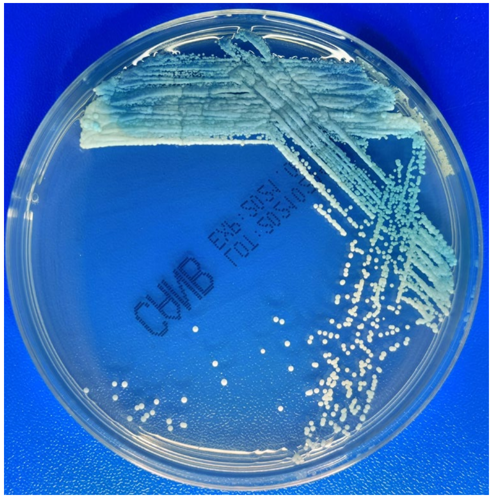

CHROM, 28°C, 72h

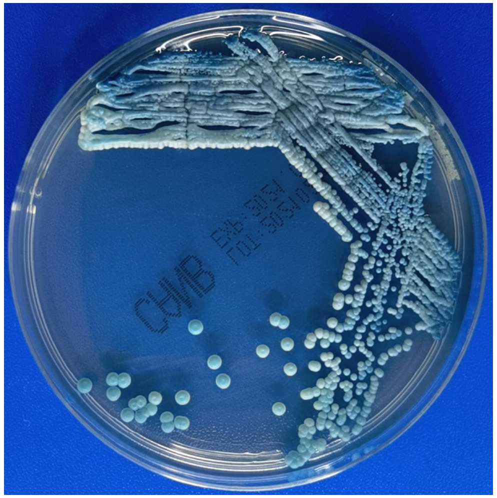

CHROM, 28°C,7d

S-12

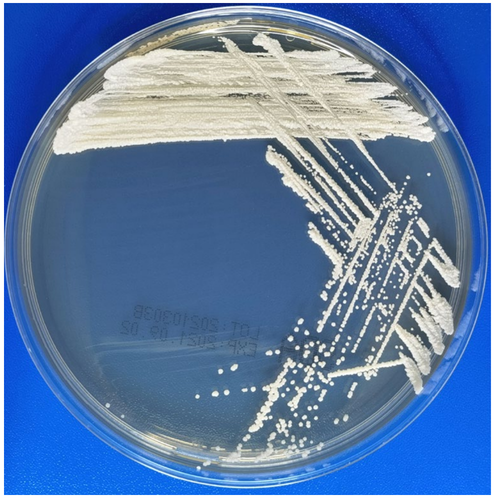

SDA, 28°C,72h

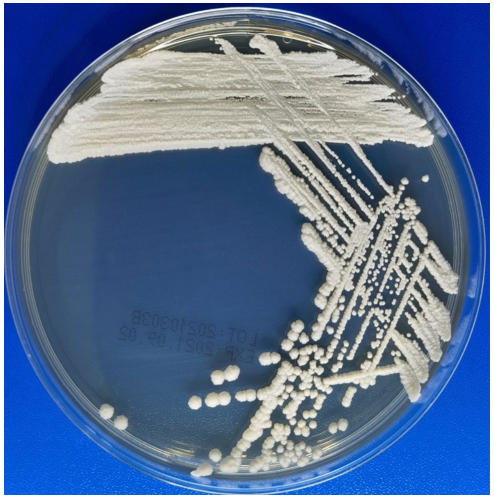

SDA, 28°C,7d

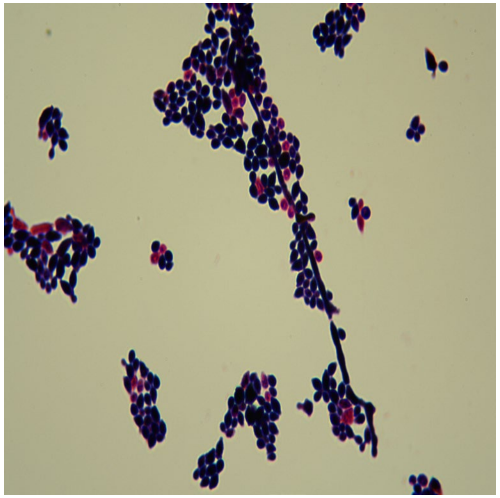

Gram staining

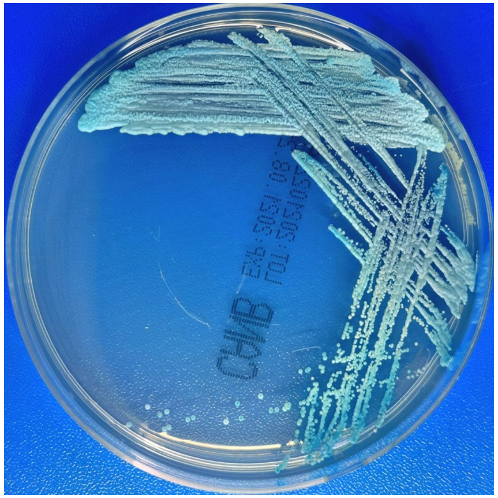

CHROM, 28°C, 72h

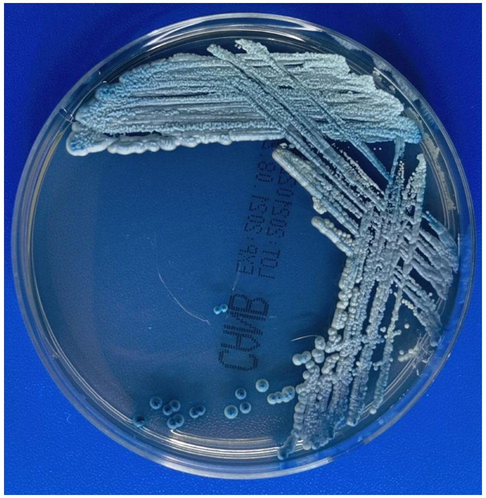

CHROM, 28°C,7d

S-13

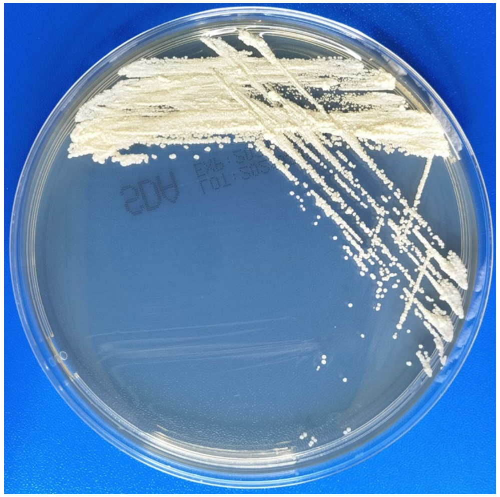

SDA, 28°C,72h

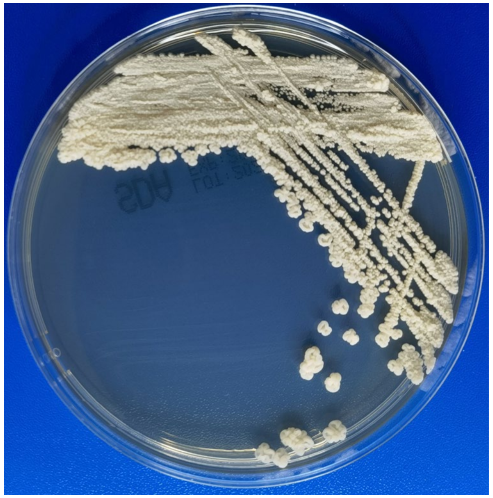

SDA, 28°C,7d

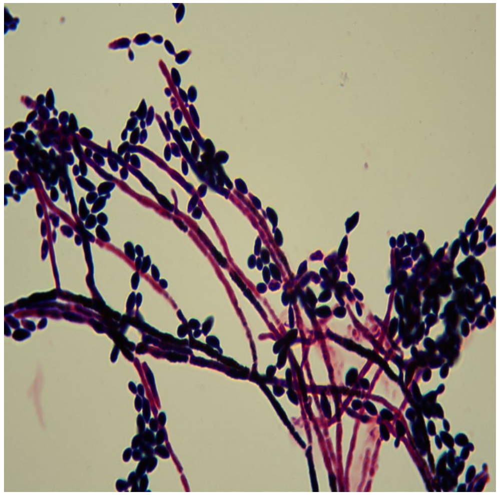

Gram staining

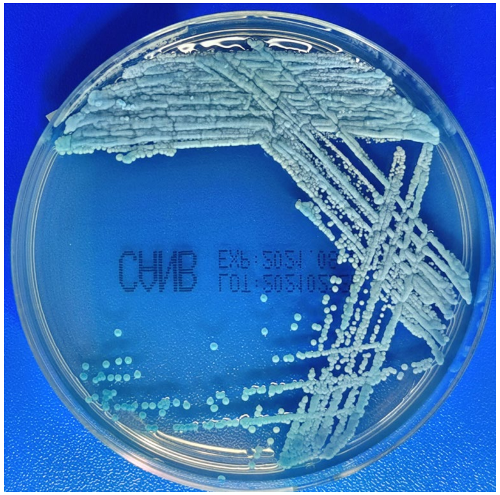

CHROM, 28°C, 72h

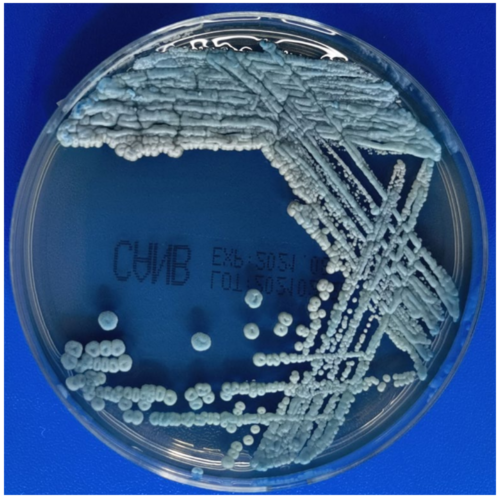

CHROM, 28°C,7d

S-14

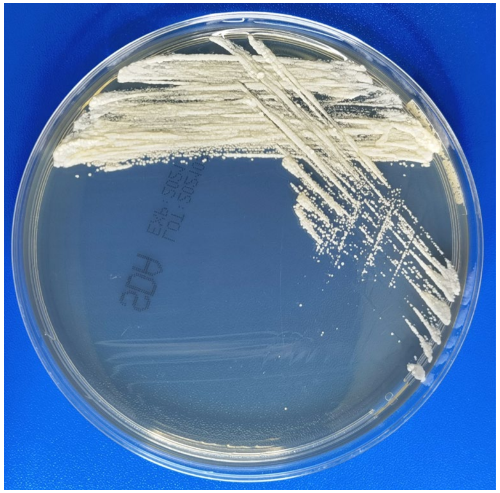

SDA, 28°C,72h

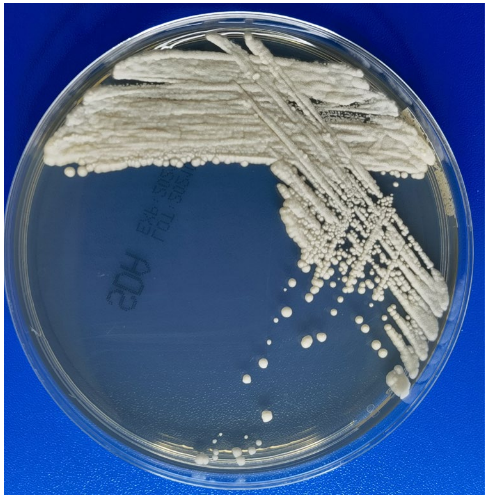

SDA, 28°C,7d

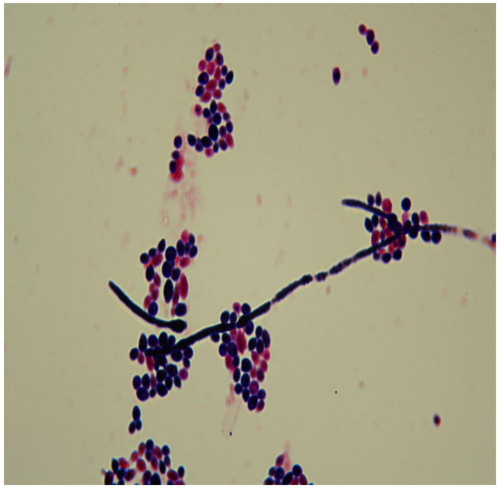

Gram staining

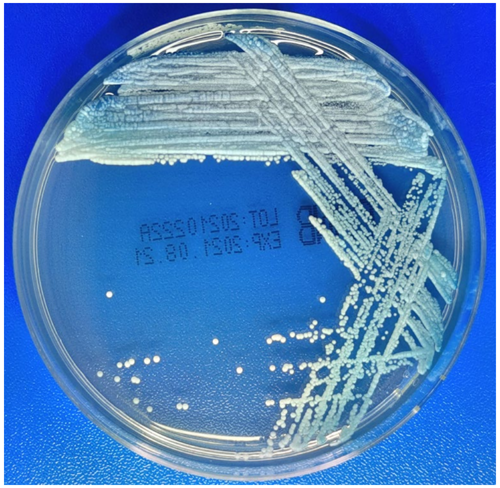

CHROM, 28°C, 72h

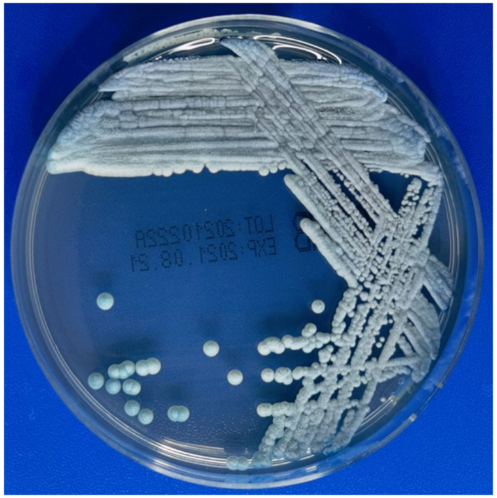

CHROM, 28°C,7d

S-15

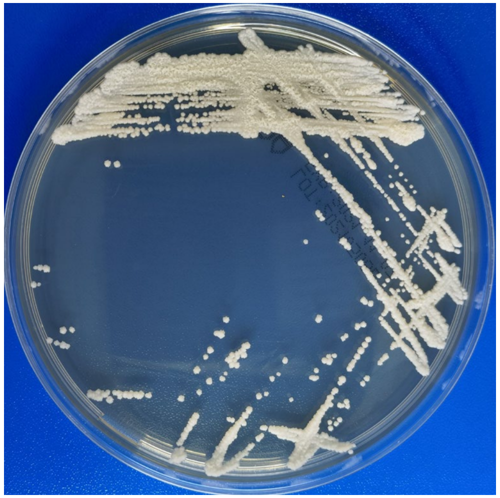

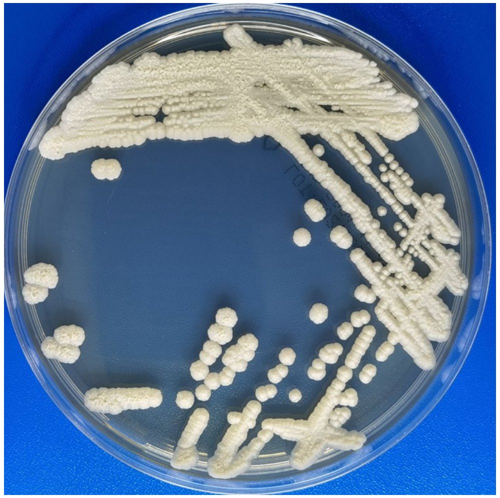

SDA, 28°C,72h

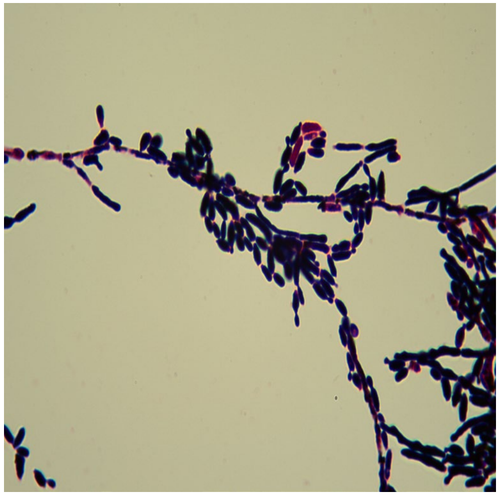

Gram staining

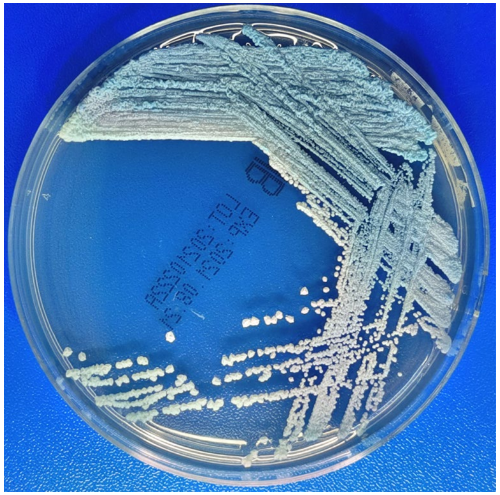

CHROM, 28°C, 72h

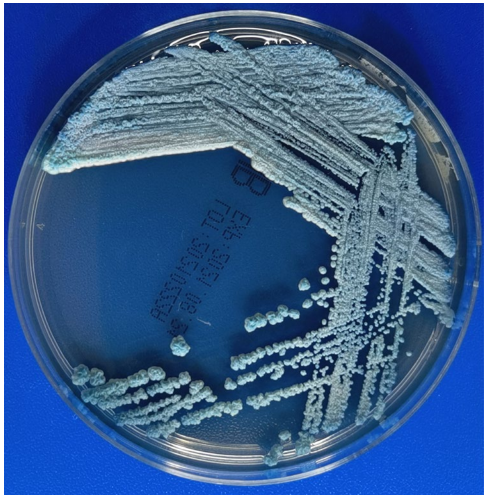

CHROM, 28°C,7d

S-16

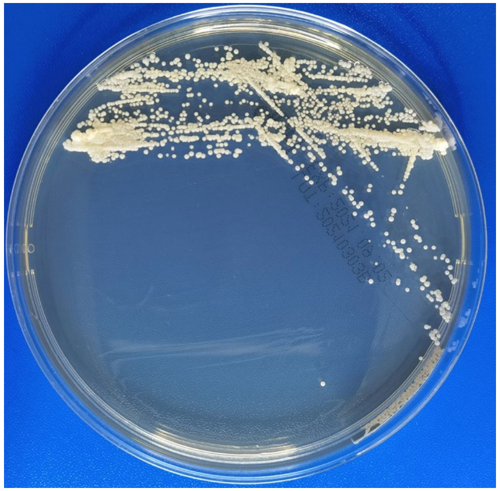

SDA, 28°C,72h

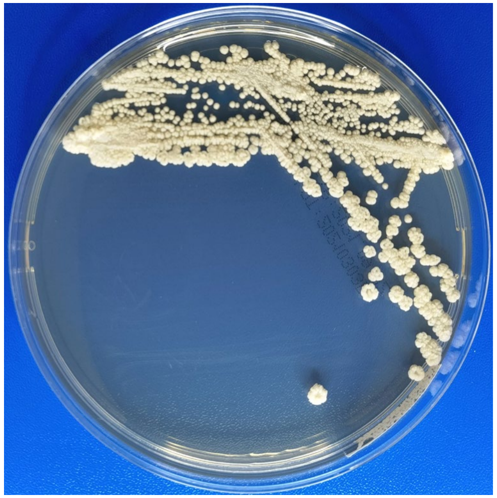

SDA, 28°C,7d

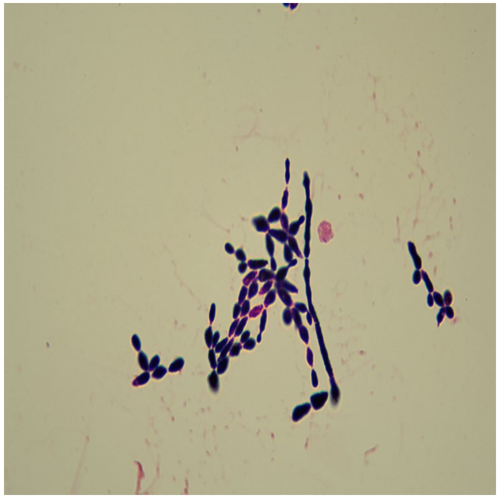

Gram staining

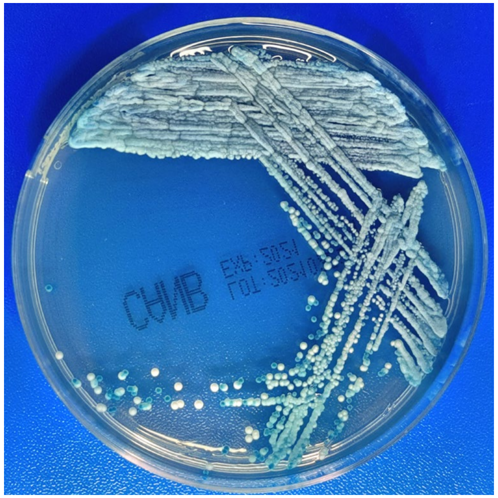

CHROM, 28°C, 72h

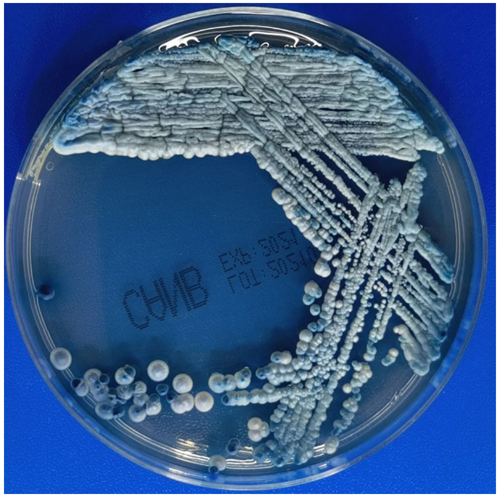

CHROM, 28°C,7d

S-17

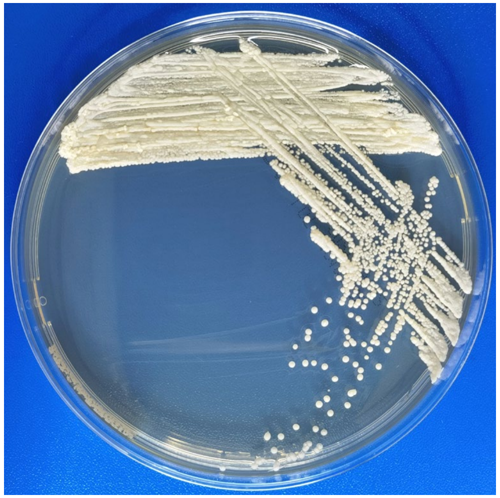

SDA, 28°C,72h

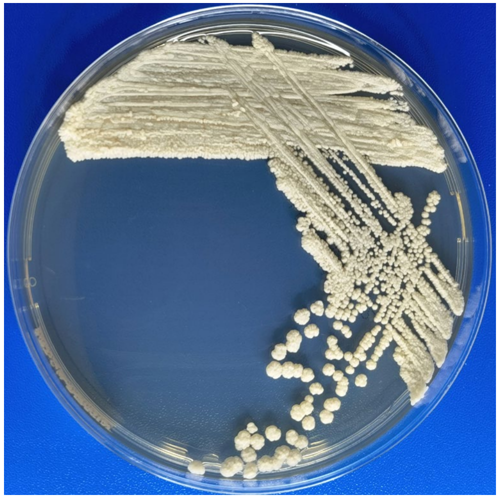

SDA, 28°C,7d

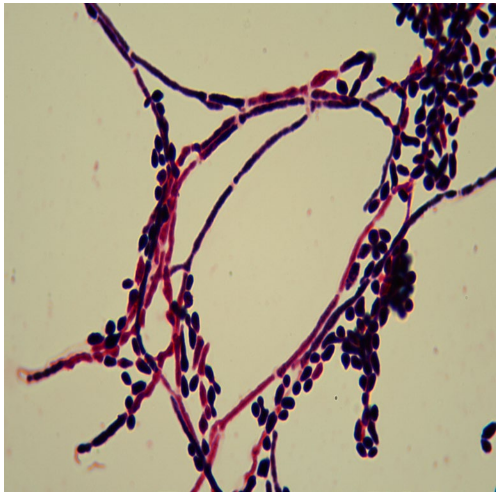

Gram staining

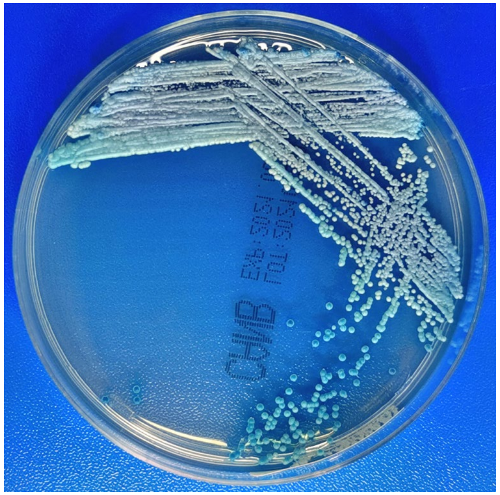

CHROM, 28°C, 72h

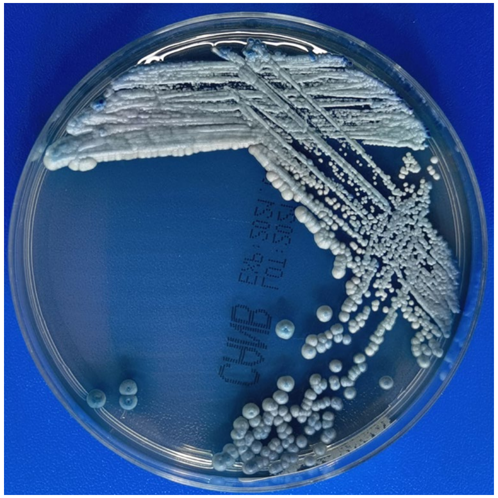

CHROM, 28°C,7d

S-18

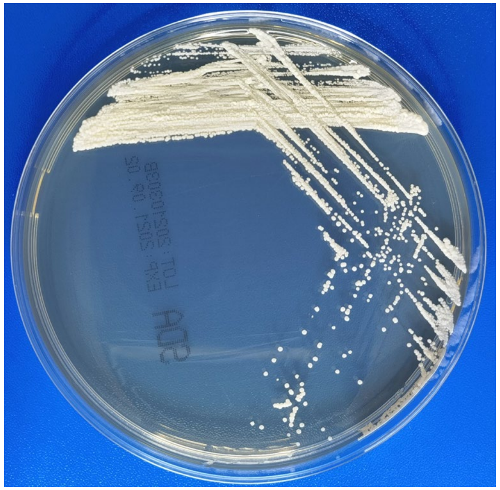

SDA, 28°C,72h

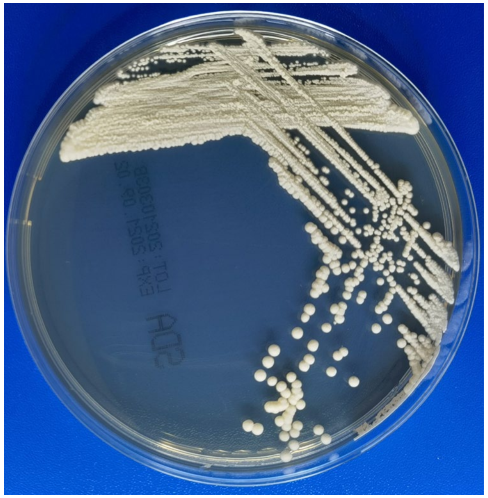

SDA, 28°C,7d

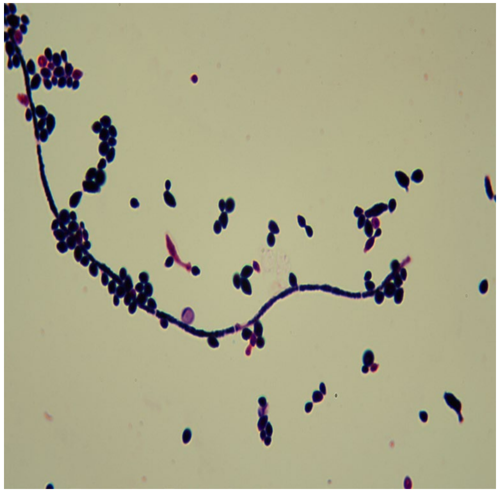

Gram staining

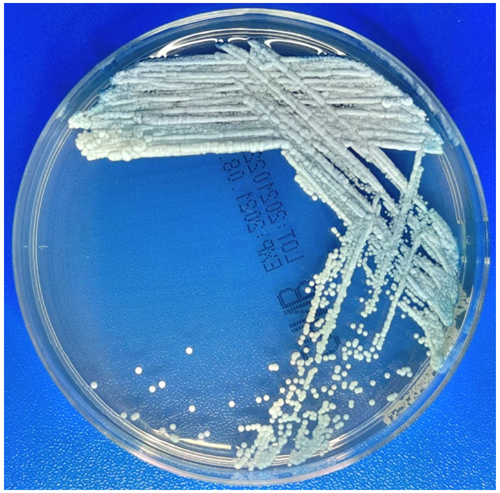

CHROM, 28°C, 72h

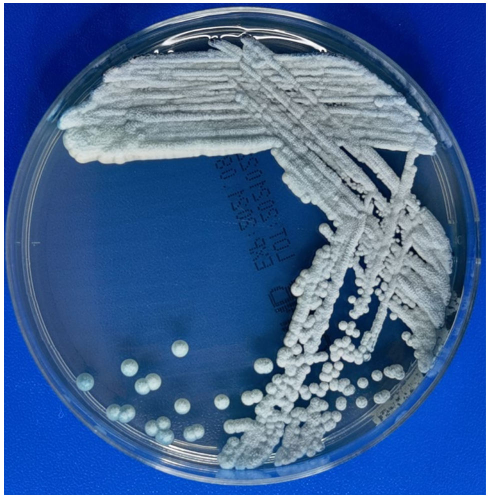

CHROM, 28°C,7d

S-19

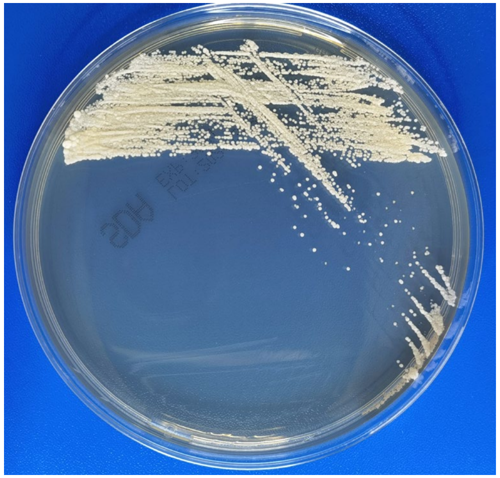

SDA, 28°C,72h

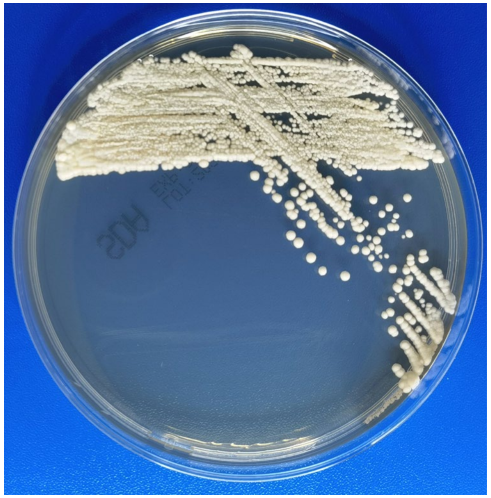

SDA, 28°C,7d

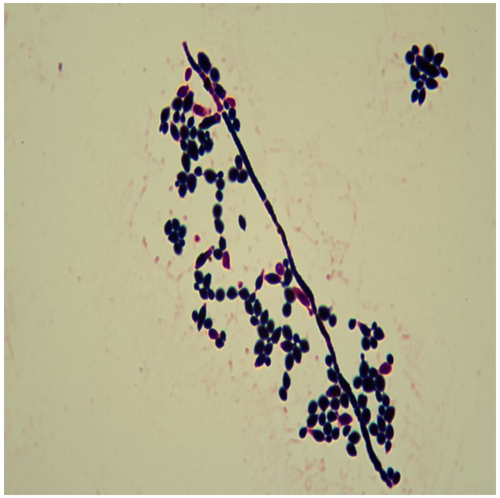

Gram staining

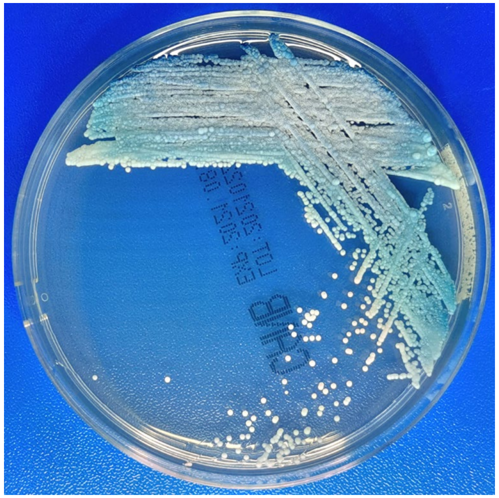

CHROM, 28°C, 72h

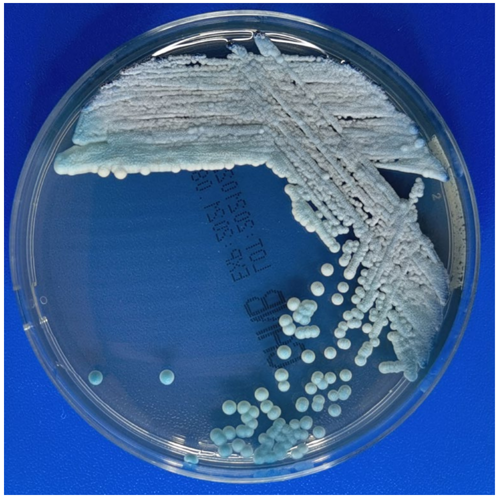

CHROM, 28°C,7d

S-20

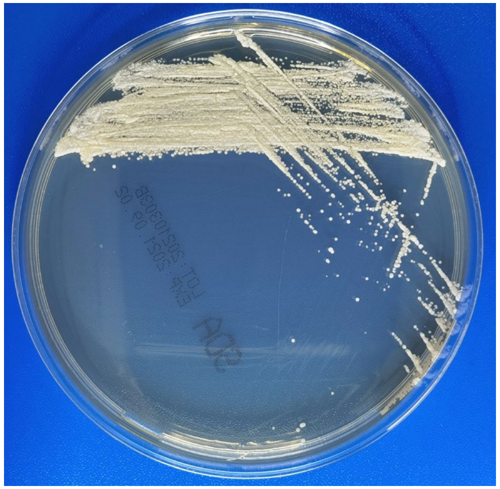

SDA, 28°C,72h

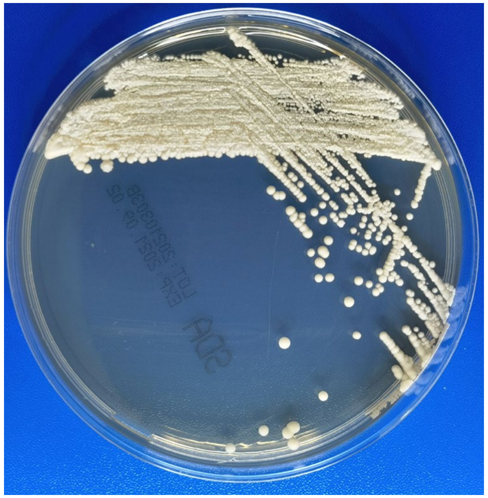

SDA, 28°C,7d

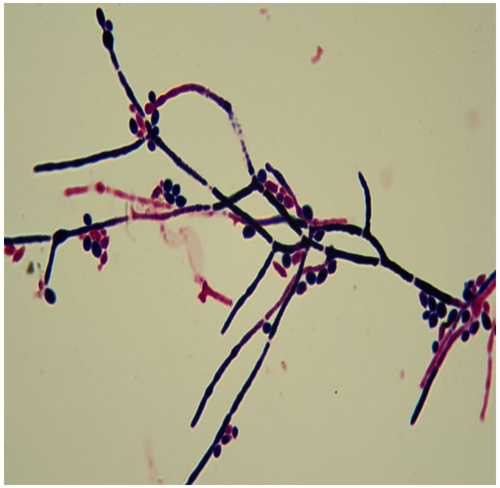

Gram staining

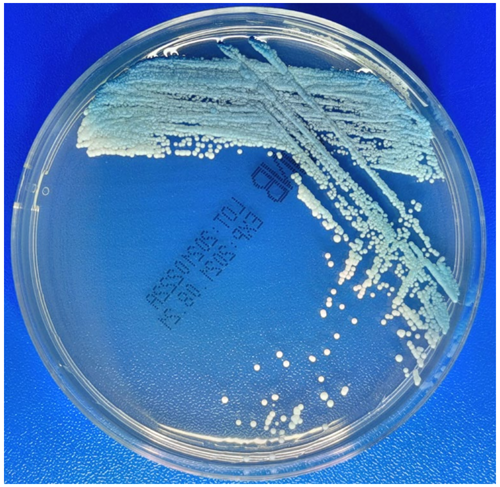

CHROM, 28°C, 72h

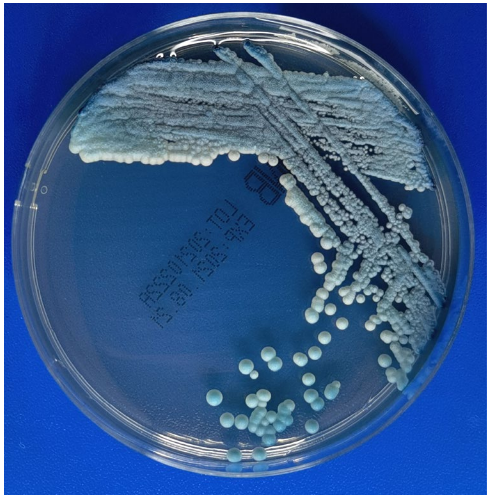

CHROM, 28°C,7d

S-21

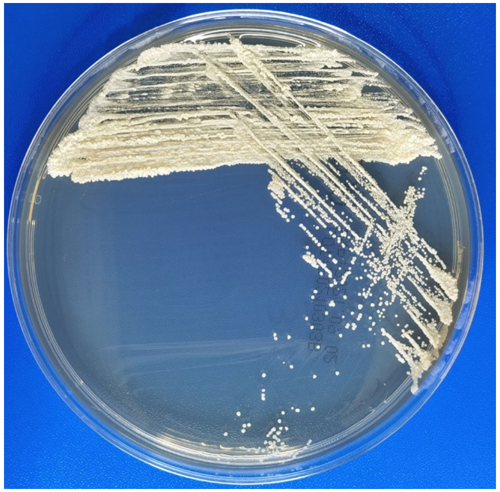

SDA, 28°C,72h

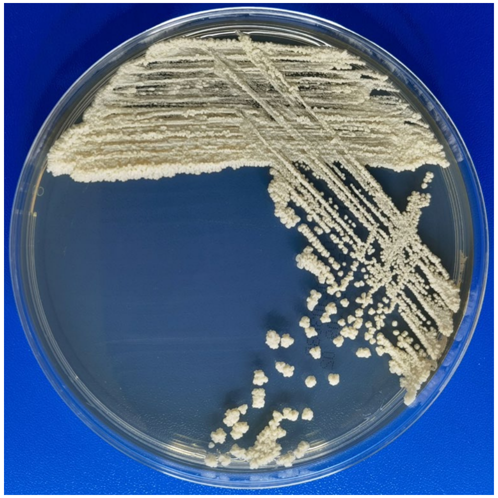

SDA, 28°C,7d

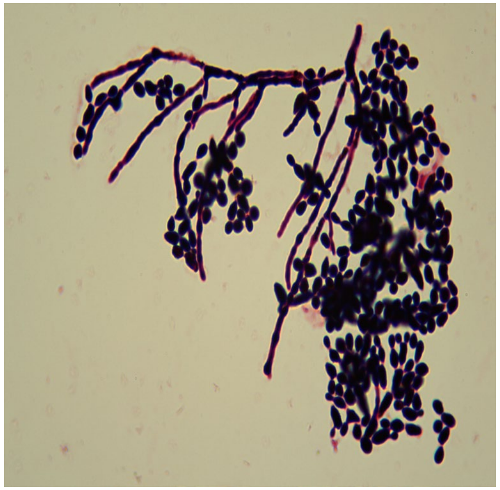

Gram staining

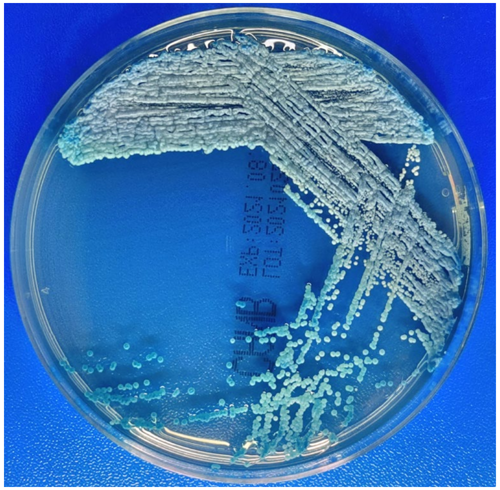

CHROM, 28°C, 72h

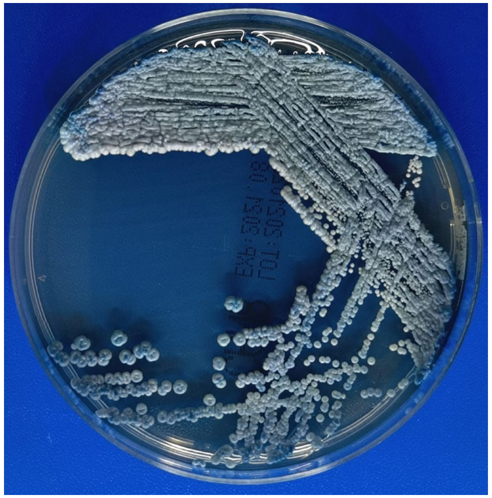

CHROM, 28°C,7d

S-22

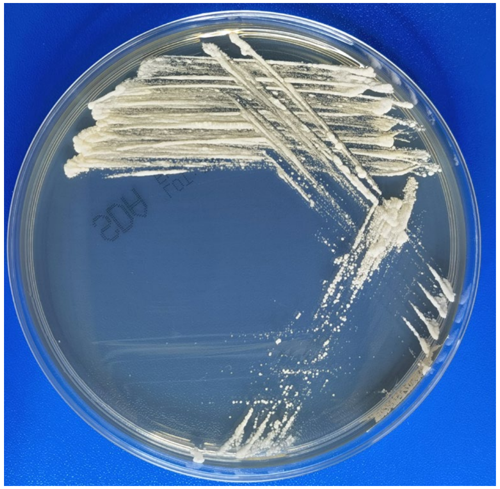

SDA, 28°C,72h

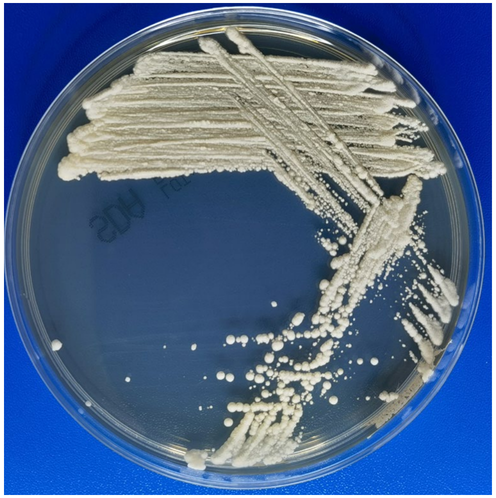

SDA, 28°C,7d

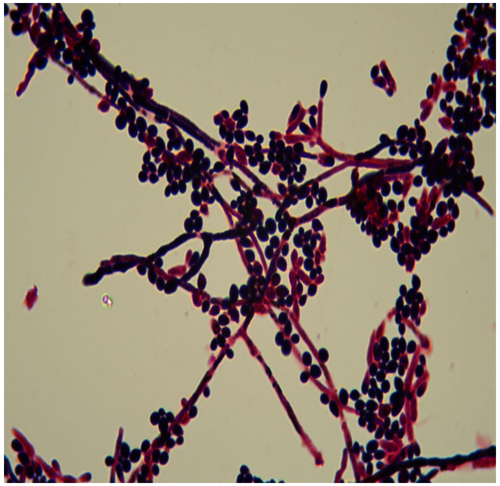

Gram staining

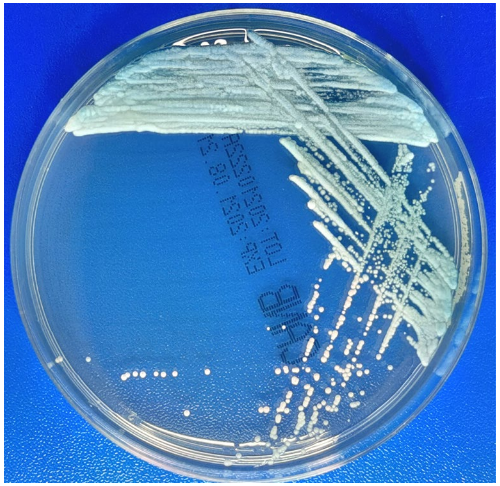

CHROM, 28°C, 72h

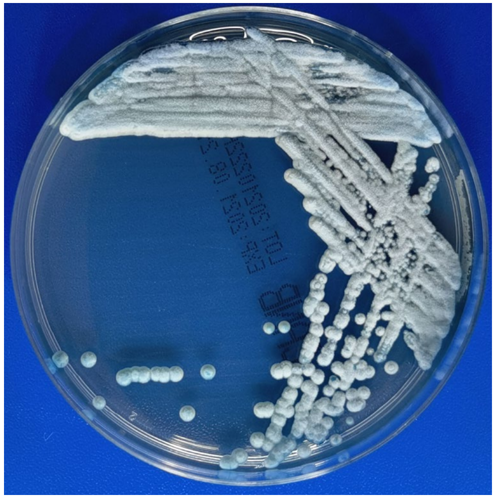

CHROM, 28°C,7d

S-23

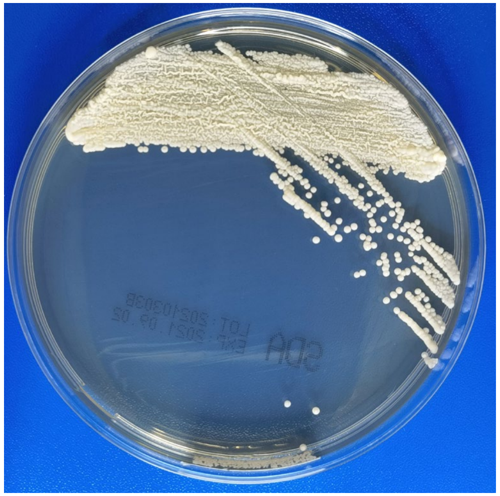

SDA, 28°C,72h

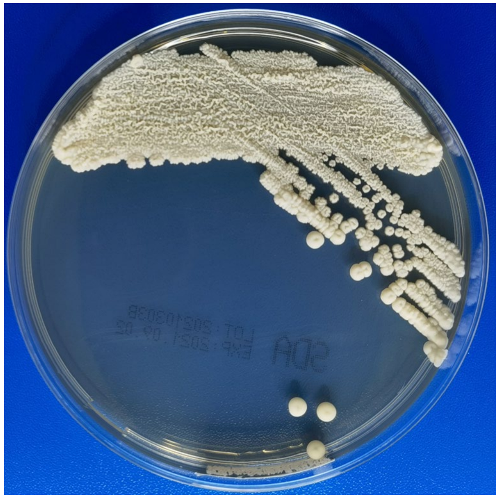

SDA, 28°C,7d

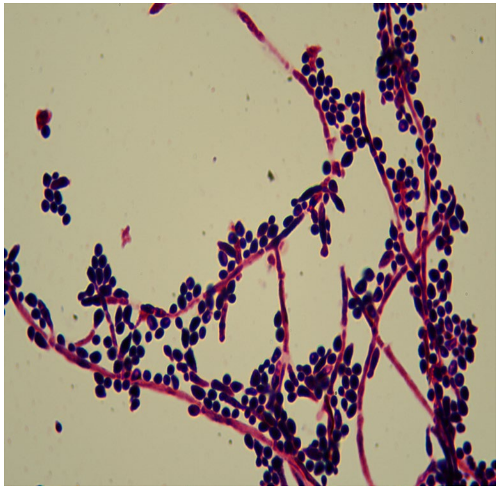

Gram staining

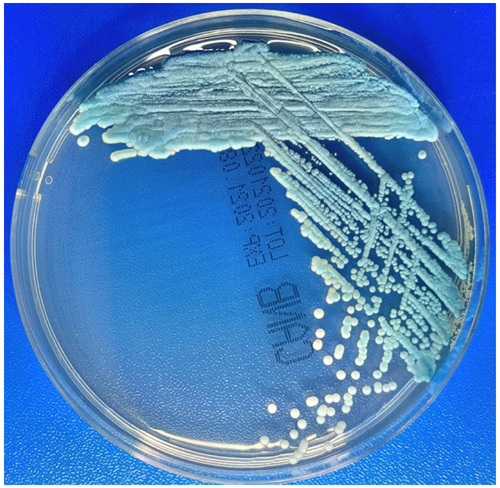

CHROM, 28°C, 72h

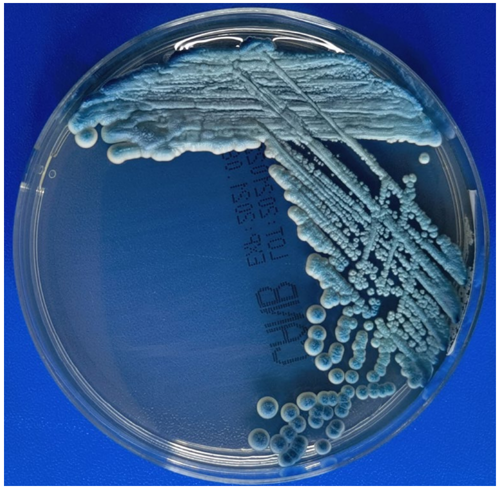

CHROM, 28°C,7d

S-24

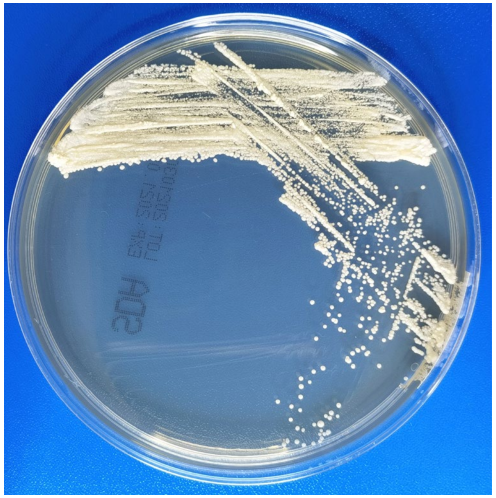

SDA, 28°C,72h

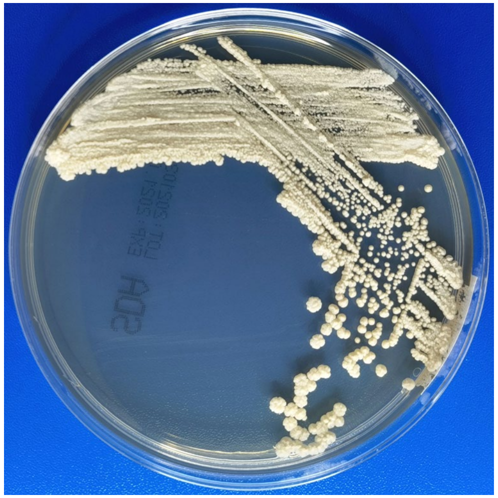

SDA, 28°C,7d

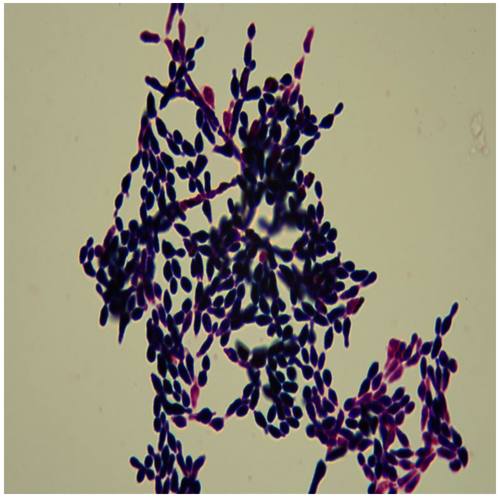

Gram staining

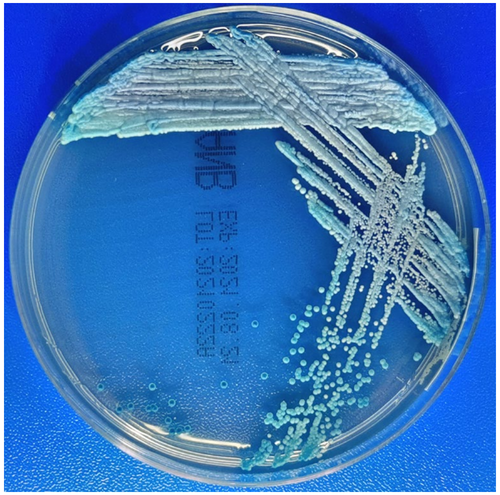

CHROM, 28°C, 72h

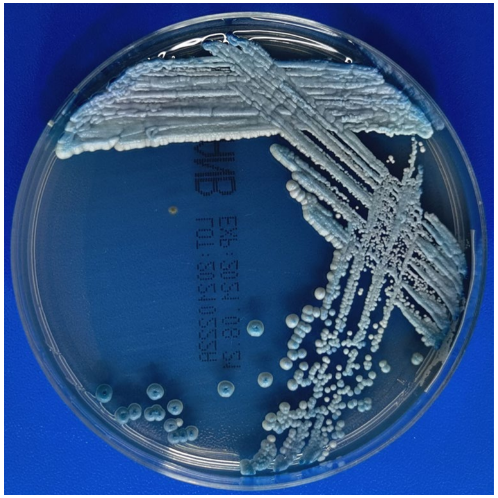

CHROM, 28°C,7d

S-25

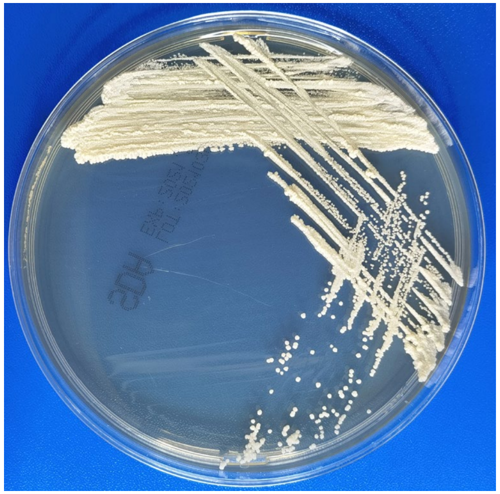

SDA, 28°C,72h

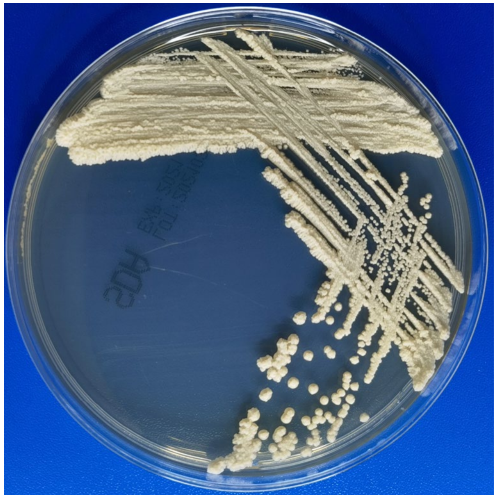

SDA, 28°C,7d

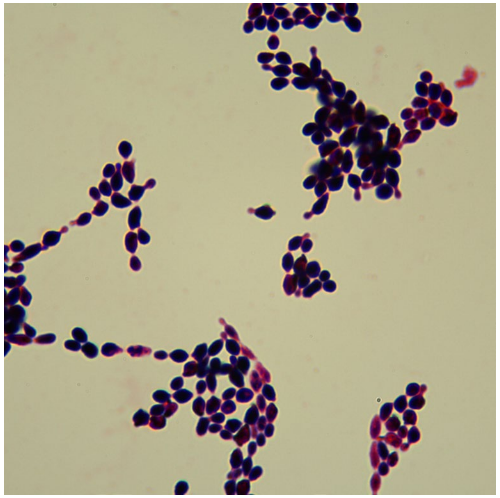

Gram staining

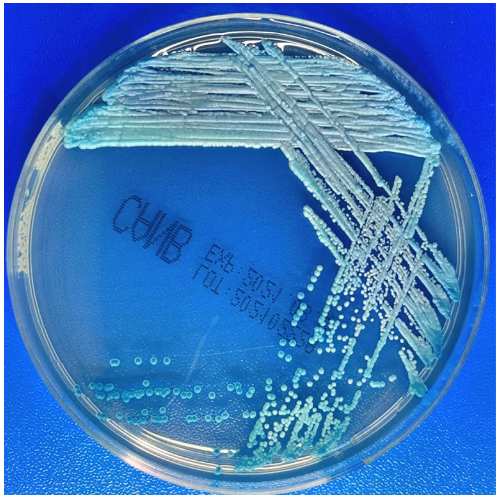

CHROM, 28°C, 72h

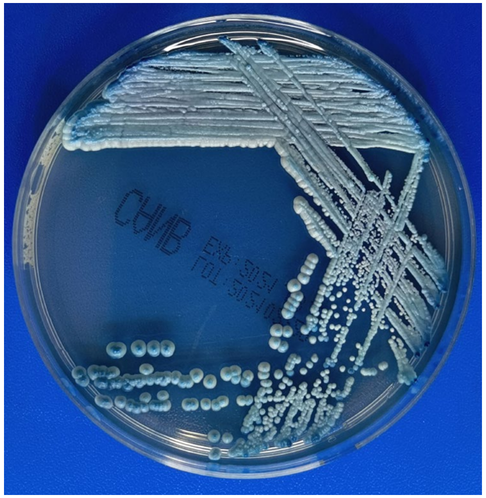

CHROM, 28°C,7d

S-26

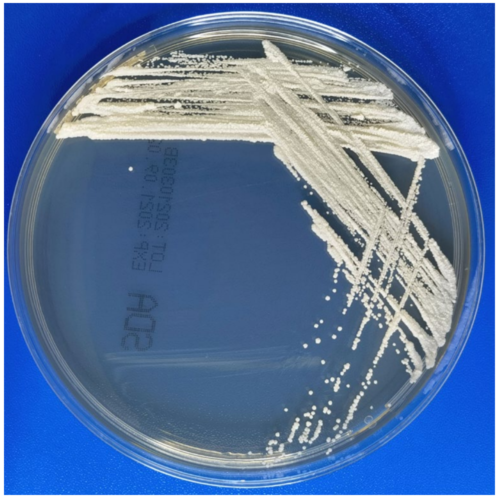

SDA, 28°C,72h

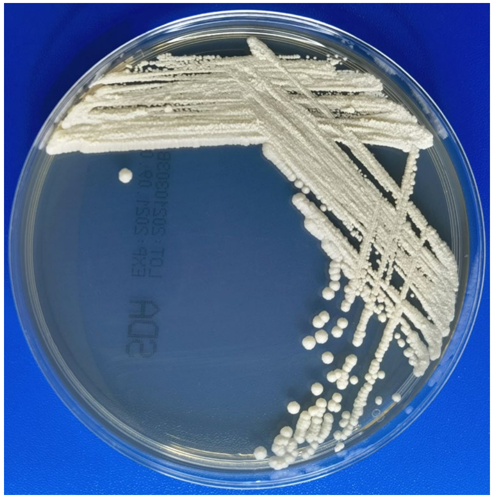

SDA, 28°C,7d

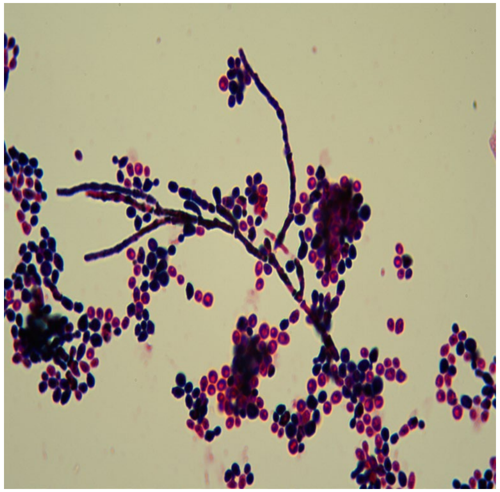

Gram staining

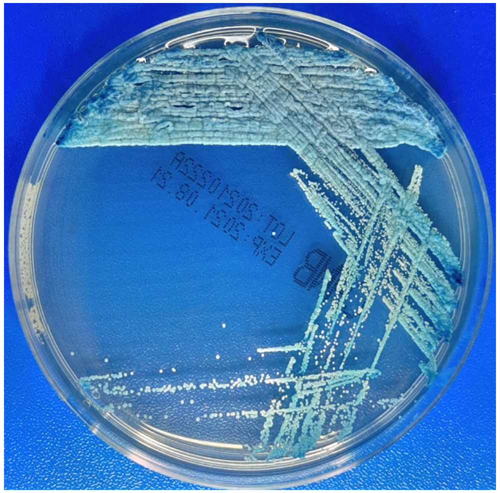

CHROM, 28°C, 72h

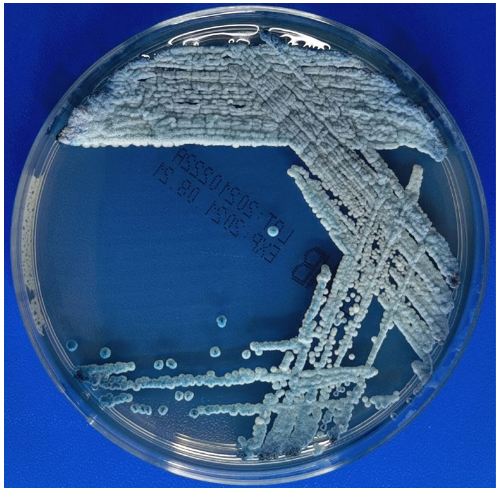

CHROM, 28°C,7d

S-27

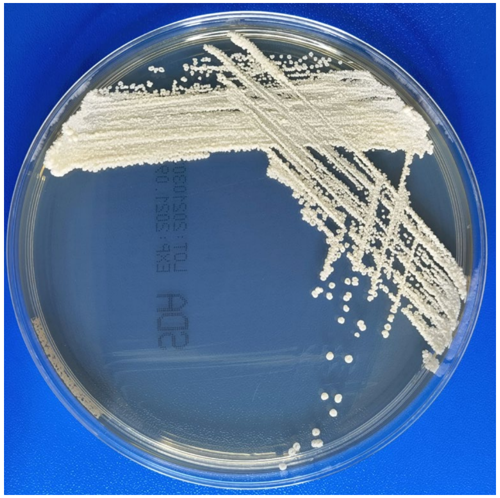

SDA, 28°C,72h

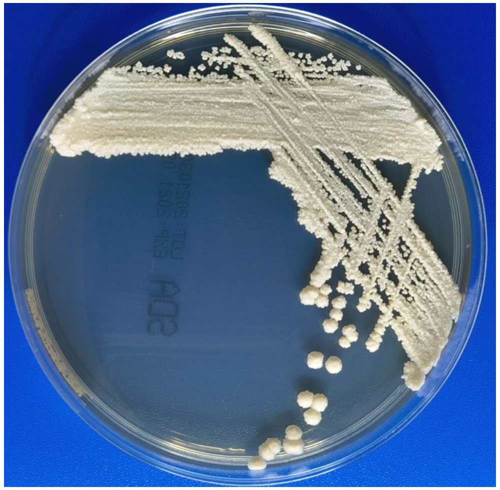

SDA, 28°C,7d

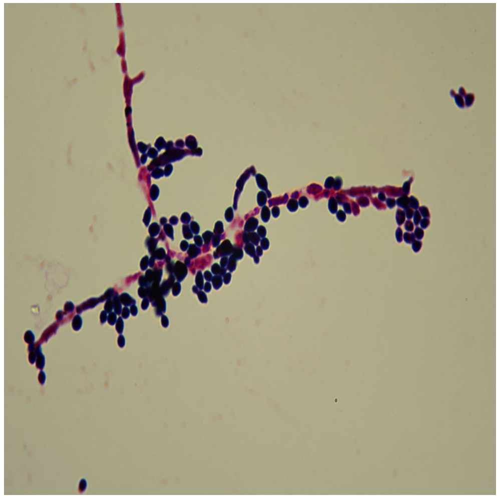

Gram staining

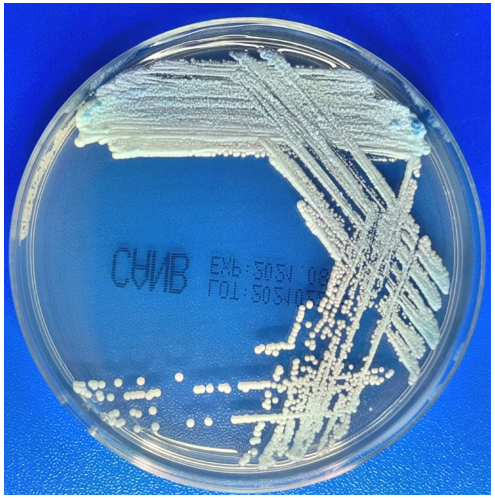

CHROM, 28°C, 72h

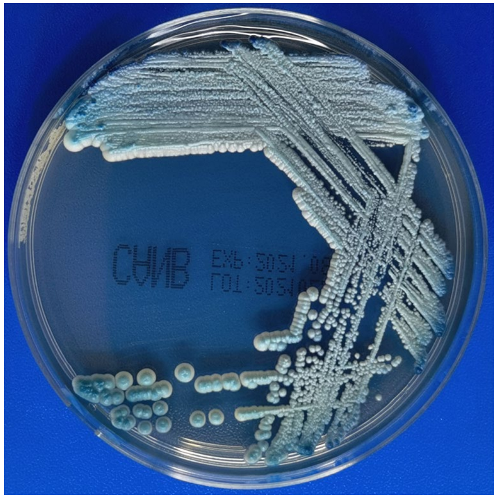

CHROM, 28°C,7d

S-28

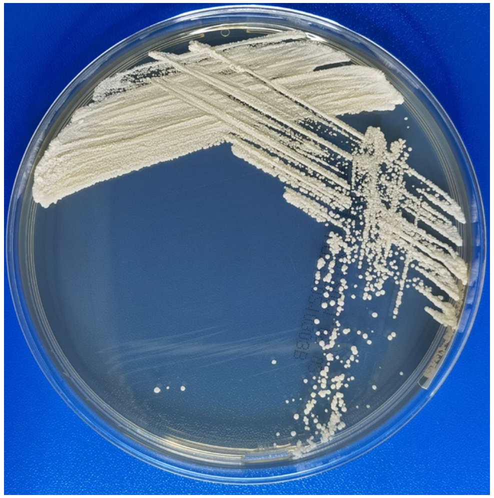

SDA, 28°C,72h

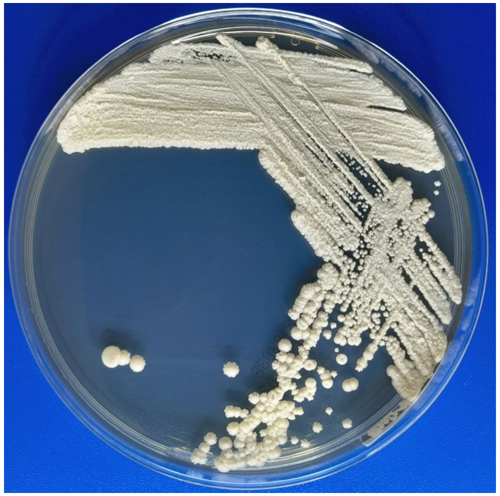

SDA, 28°C,7d

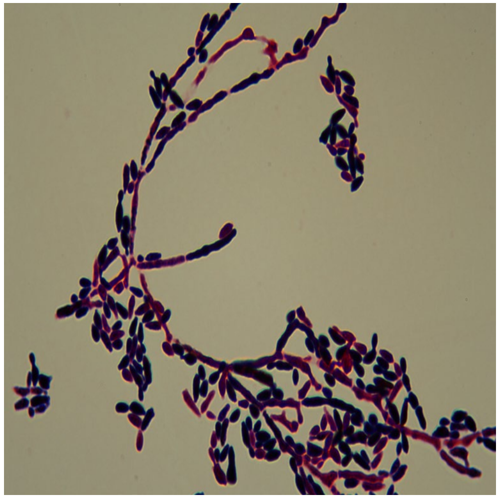

Gram staining

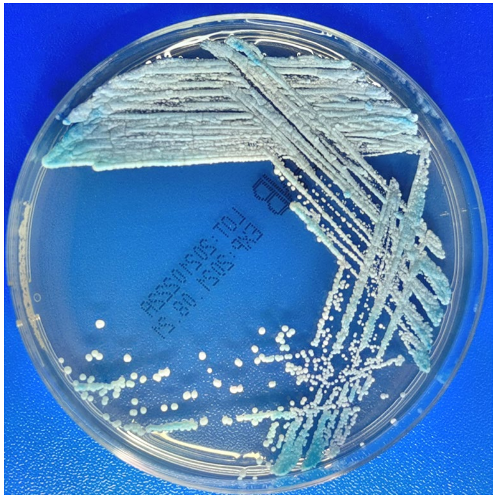

CHROM, 28°C, 72h

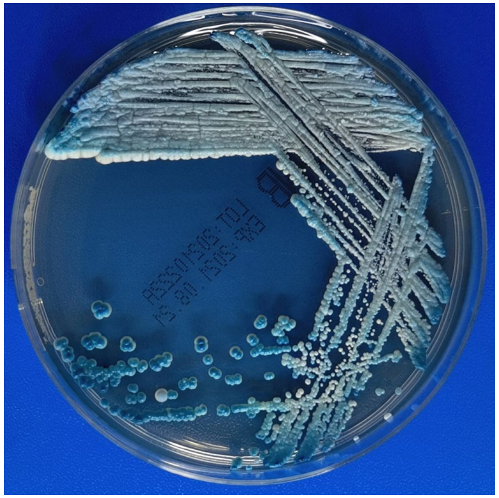

CHROM, 28°C,7d

S-29

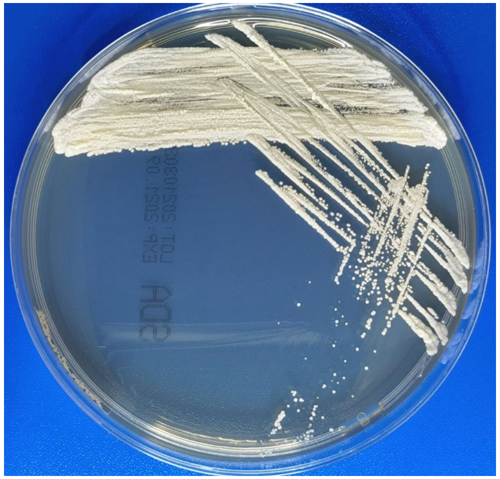

SDA, 28°C,72h

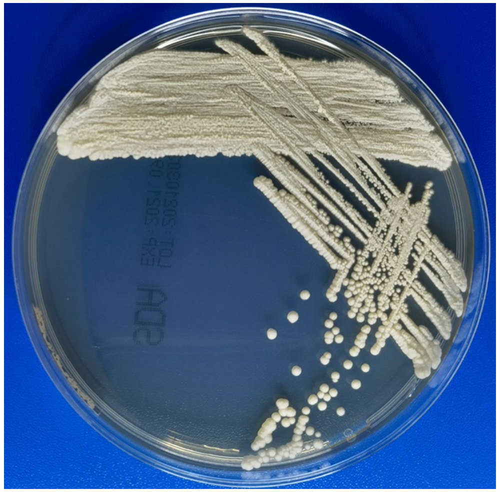

SDA, 28°C,7d

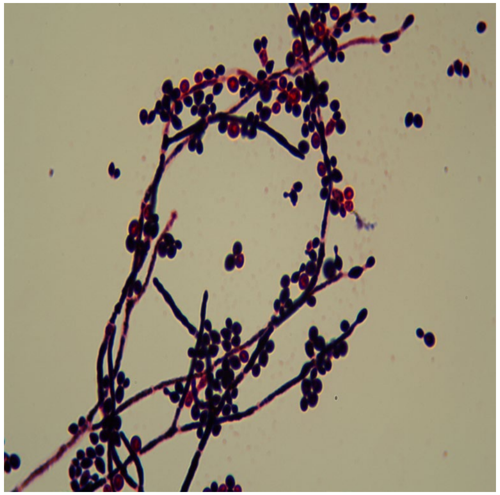

Gram staining

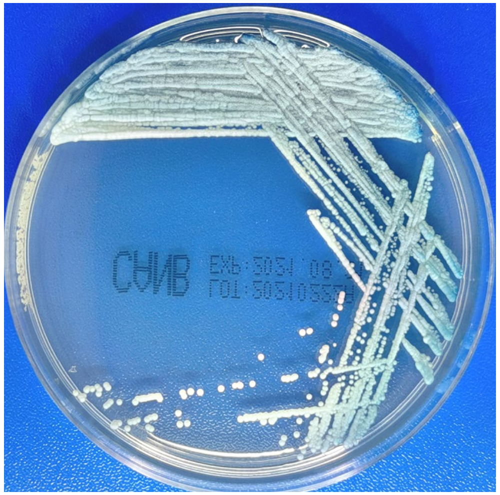

CHROM, 28°C, 72h

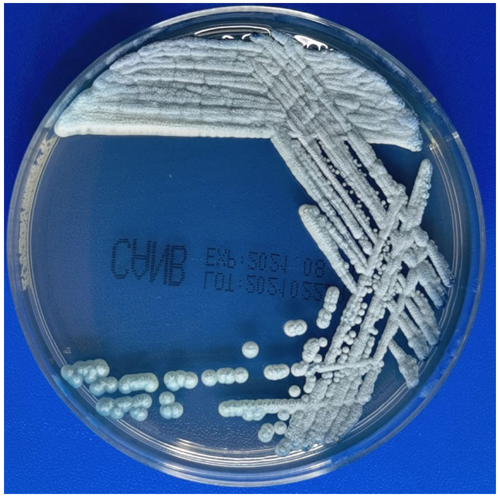

CHROM, 28°C,7d

S-30

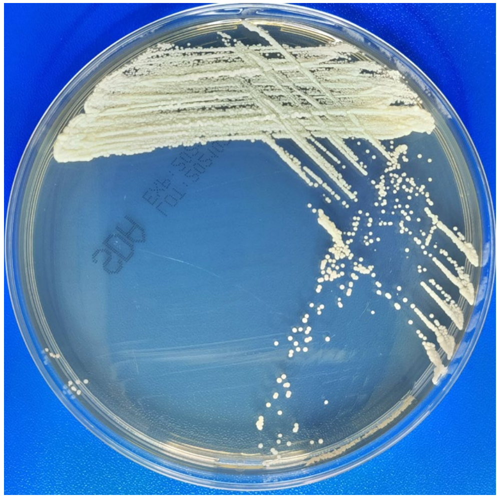

SDA, 28°C,72h

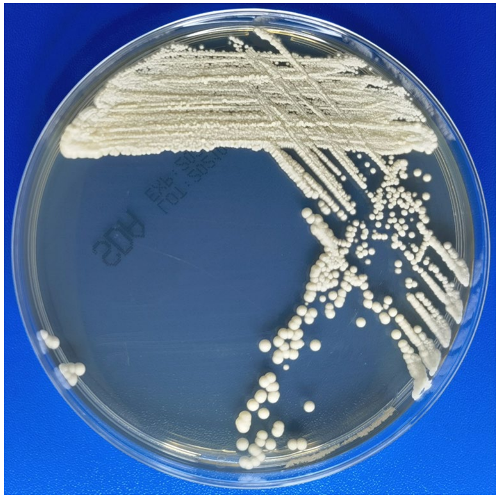

SDA, 28°C,7d

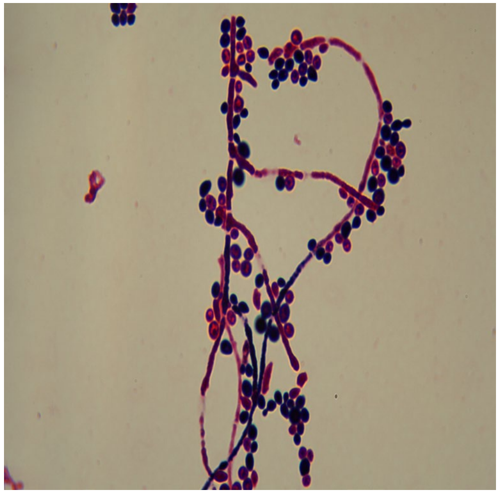

Gram staining

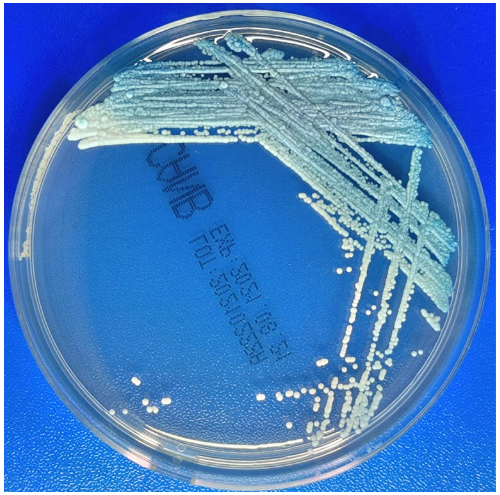

CHROM, 28°C, 72h

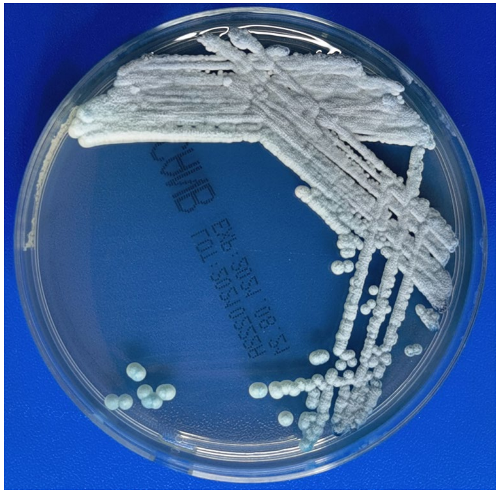

CHROM, 28°C,7d

S-31

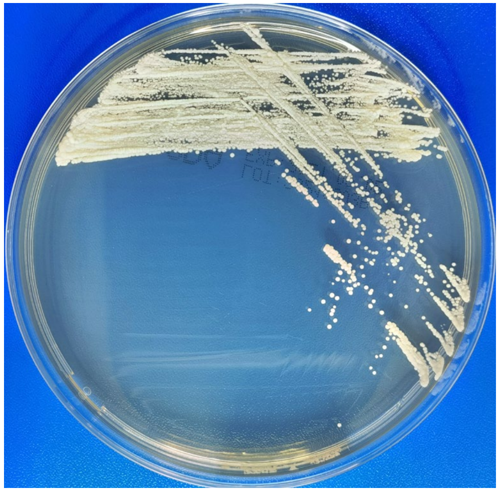

SDA，28℃,72h

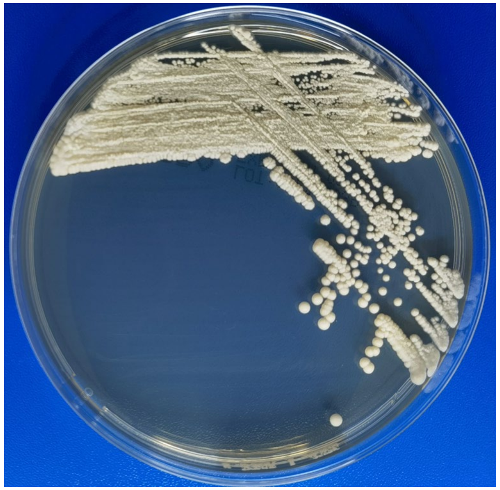

SDA，28℃,7d

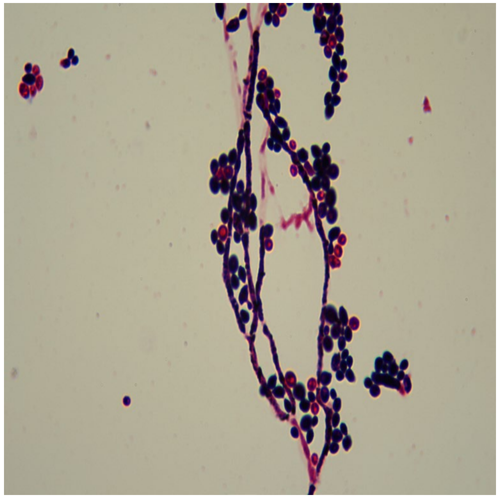

Gram staining

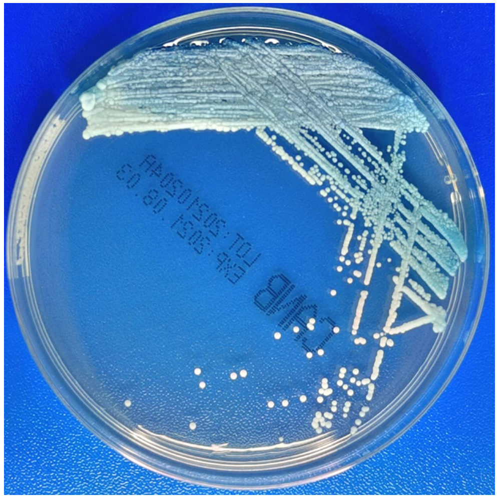

CHROM，28℃, 72h

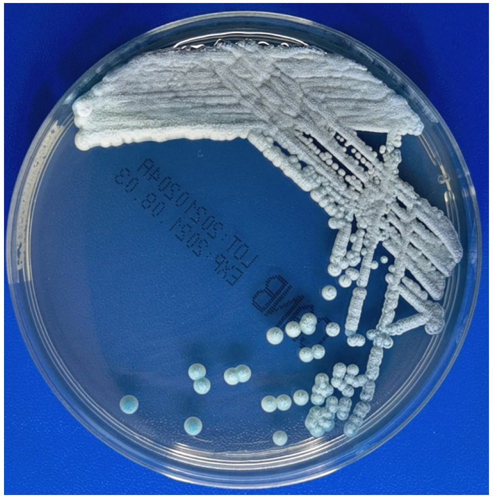

CHROM，28℃,7d

S-32

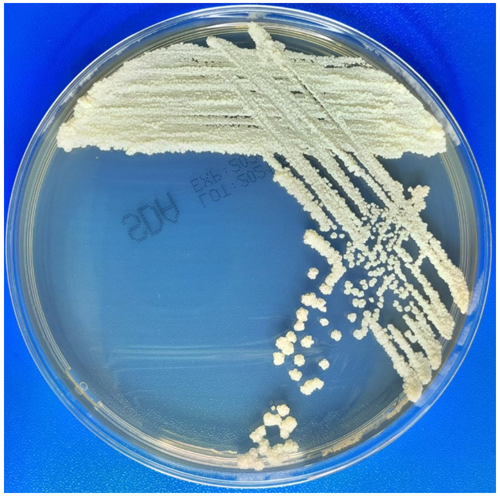

SDA, 28°C,72h

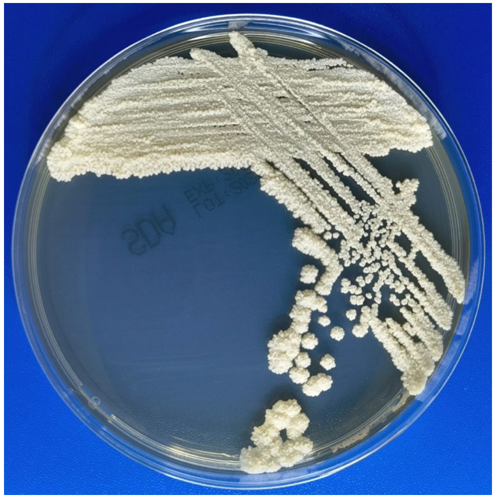

SDA, 28°C,7d

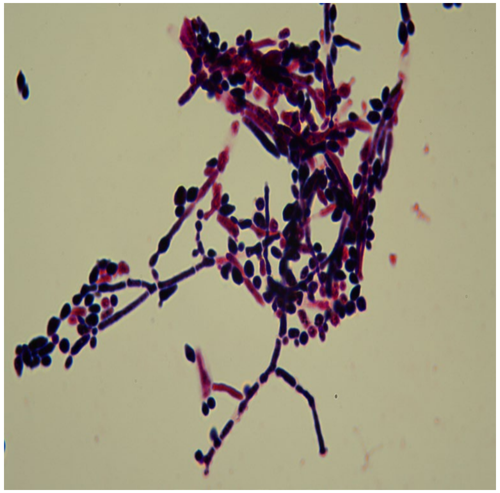

Gram staining

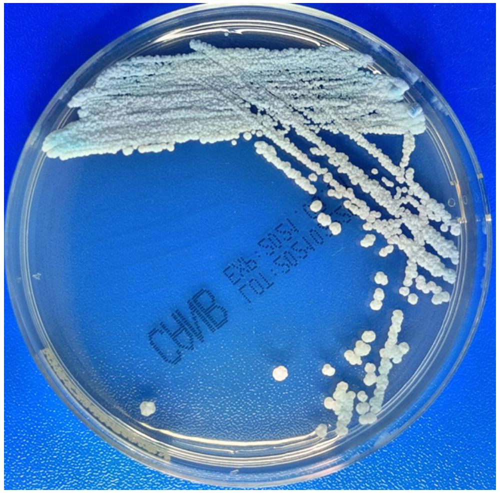

CHROM, 28°C, 72h

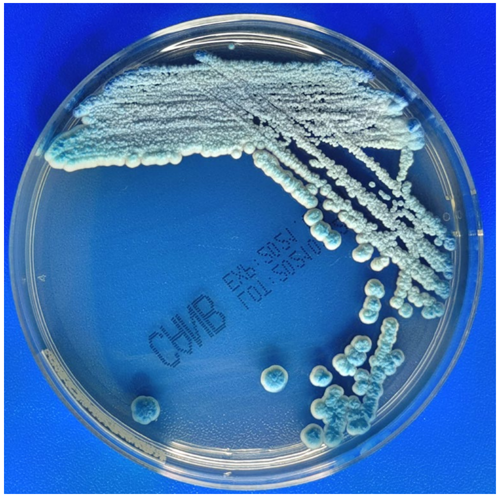

CHROM, 28°C,7d
